# Comparison of Outcomes before and after Ohio's Law Mandating Use of the FDA-Approved Protocol for Medication Abortion: A Retrospective Cohort Study

**DOI:** 10.1371/journal.pmed.1002110

**Published:** 2016-08-30

**Authors:** Ushma D. Upadhyay, Nicole E. Johns, Sarah L. Combellick, Julia E. Kohn, Lisa M. Keder, Sarah C. M. Roberts

**Affiliations:** 1 Advancing New Standards in Reproductive Health (ANSIRH), Bixby Center for Global Reproductive Health, Department of Obstetrics, Gynecology and Reproductive Sciences, University of California, San Francisco, Oakland, California, United States of America; 2 Planned Parenthood Federation of America, New York, New York, United States of America; 3 Obstetrics and Gynecology, Ohio State University Wexner Medical Center, Ohio State University, Columbus, Ohio, United States of America; University of Toronto, CANADA

## Abstract

**Background:**

In February 2011, an Ohio law took effect mandating use of the United States Food and Drug Administration (FDA)-approved protocol for mifepristone, which is used with misoprostol for medication abortion. Other state legislatures have passed or enacted similar laws requiring use of the FDA-approved protocol for medication abortion. The objective of this study is to examine the association of this legal change with medication abortion outcomes and utilization.

**Methods and Findings:**

We used a retrospective cohort design, comparing outcomes of medication abortion patients in the prelaw period to those in the postlaw period. Sociodemographic and clinical chart data were abstracted from all medication abortion patients from 1 y prior to the law’s implementation (January 2010–January 2011) to 3 y post implementation (February 2011–October 2014) at four abortion-providing health care facilities in Ohio. Outcome data were analyzed for all women undergoing abortion at ≤49 d gestation during the study period. The main outcomes were as follows: need for additional intervention following medication abortion (such as aspiration, repeat misoprostol, and blood transfusion), frequency of continuing pregnancy, reports of side effects, and the proportion of abortions that were medication abortions (versus other abortion procedures). Among the 2,783 medication abortions ≤49 d gestation, 4.9% (95% CI: 3.7%–6.2%) in the prelaw and 14.3% (95% CI: 12.6%–16.0%) in the postlaw period required one or more additional interventions. Women obtaining a medication abortion in the postlaw period had three times the odds of requiring an additional intervention as women in the prelaw period (adjusted odds ratio [AOR] = 3.11, 95% CI: 2.27–4.27). In a mixed effects multivariable model that uses facility-months as the unit of analysis to account for lack of independence by site, we found that the law change was associated with a 9.4% (95% CI: 4.0%–18.4%) absolute increase in the rate of requiring an additional intervention. The most common subsequent intervention in both periods was an additional misoprostol dose and was most commonly administered to treat incomplete abortion. The percentage of women requiring two or more follow-up visits increased from 4.2% (95% CI: 3.0%–5.3%) in the prelaw period to 6.2% (95% CI: 5.5%–8.0%) in the postlaw period (*p* = 0.003). Continuing pregnancy was rare (0.3%). Overall, 12.6% of women reported at least one side effect during their medication abortion: 8.4% (95% CI: 6.8%–10.0%) in the prelaw period and 15.6% (95% CI: 13.8%–17.3%) in the postlaw period (*p* < 0.001). Medication abortions fell from 22% (95% CI: 20.8%–22.3%) of all abortions the year before the law went into effect (2010) to 5% (95% CI: 4.8%–5.6%) 3 y after (2014) (*p* < 0.001). The average patient charge increased from US$426 in 2010 to US$551 in 2014, representing a 16% increase after adjusting for inflation in medical prices. The primary limitation to the study is that it was a pre/post-observational study with no control group that was not exposed to the law.

**Conclusions:**

Ohio law required use of a medication abortion protocol that is associated with a greater need for additional intervention, more visits, more side effects, and higher costs for women relative to the evidence-based protocol. There is no evidence that the change in law led to improved abortion outcomes. Indeed, our findings suggest the opposite. In March 2016, the FDA-protocol was updated, so Ohio providers may now legally provide current evidence-based protocols. However, this law is still in place and bans physicians from using mifepristone based on any new developments in clinical research as best practices continue to be updated.

## Abstract 2

In this retrospective cohort study, Ushma Upadhyay and colleagues compare outcomes from medication abortions performed in Ohio before and after a law mandating use of the FDA-approved protocol.

## Author Summary

**Why Was This Study Done?:**

- An Ohio law went into effect in 2011 that required abortion providers to use a protocol for medication abortion that had been approved by the US Food and Drug Administration (FDA) in 2000.
- This protocol conflicted with the protocol supported by several international guidelines and used by most abortion providers throughout the US.
- The protocol approved by the FDA in 2000 required a higher, more expensive dose of oral mifepristone, a lower dose of oral misoprostol administered only at a provider’s office 48 h later, and limited use up to 49 d after a woman’s last menstrual period.
- This research was conducted to explore the abortion outcomes for women who received medication abortion before the 2011 law went into effect compared with outcomes after the law was in place.

**What Did the Researchers Do and Find?:**

- Using chart data from 2,783 women who obtained a medication abortion between 2010 and 2014 collected retrospectively from four clinics in Ohio, we examined the proportion of women who received an additional medical intervention to complete the abortion, the experience of side effects, and the rate of medication abortion versus aspiration abortion in Ohio.
- The data showed that women who had medication abortions in the postlaw period were three times as likely to need additional interventions to complete their abortion compared to women in the prelaw period.
- In addition, side effects such as nausea and vomiting were significantly more likely among women after the law change, and there was an 80% decline in medication abortion in Ohio between 2010 and 2014.

**What Do These Findings Mean?:**

- These results suggest that after the 2011 law was enacted, there was a greater need for additional intervention, as well as more visits, more side effects, and higher costs for women compared to before the law.
- Although the FDA updated the medication abortion protocol in March 2016 to match the evidence, this protocol may also become outdated in the future, and health providers in Ohio will be required to provide care based on legislation, rather than the most up-to-date research and evidence-based practice.

## Introduction

Medication abortion is a nonsurgical abortion in which two medications are taken to induce an abortion. Mifeprex is the brand name for the drug mifepristone (previously called RU-486). Mifepristone is used in combination with misoprostol for a medication abortion. While it is sometimes called a medical abortion, we use the term medication abortion because it most accurately represents the use of drug-based methods that can terminate pregnancy [1].

The US Food and Drug Administration (FDA) first approved the sale of Mifeprex for medication abortion in September 2000 after a lengthy and charged political process, 54 mo after the application was first submitted [2,3]. The regimen that was approved specified 600 mg of oral mifepristone followed 2 d later by 400 mcg of misoprostol taken orally in a provider’s office within the first 49 d after a woman’s last menstrual period (Table 1). Research showed this regimen to be very safe [4,5], and at the time the application was first submitted, it had the most published clinical evidence to support it. However, as early as the mid-1980s, investigators were examining modifications to this initially approved regimen [6–9]. A large body of research has developed on alternative dosages of the drugs and timing and route of administration to improve success rates for medication abortion.

**Table 1:** Protocol comparison.

	Evidence-Based Regimen	Original FDA-Approved Regimen (as Approved in 2000)
Dates in Use in Ohio	Up to Jan 2011	Feb 2011 to Mar 2016
Maximum Days Gestation	63 d from LMP	49 d from LMP
Mifepristone Dose	200 mg orally in office	600 mg orally in office
Misoprostol Dose	800 mcg vaginally or buccally (4 tablets)	400 mcg orally (2 tablets)
Misoprostol Timing	6–72 h after mifepristone	48 h after mifepristone
Misoprostol Location	Home	Provider’s office
Follow-up Visit	5–14 d after mifepristone	14 d after mifepristone
Cost	Lower	Higher
Minimum Number of Office Visits (Including Ohio’s Required Information Visit)	3	4

LMP, last menstrual period. Adapted from the American College of Obstetricians and Gynecologists [18].

Thus, since 2000 (and even before [2]), abortion providers have been employing alternative regimens for medication abortions, based on the published literature [10–12]. These evidence-based regimens include a lower, less expensive 200 mg dose of oral mifepristone and a higher 800 mcg dose of misoprostol administered buccally or vaginally at home (Table 1). These regimens also allow extending use up to 63 d after a woman’s last menstrual period [13,14] and, more recently, up to 70 d, although effectiveness rates decline with increasing gestation [15–17]. At equivalent gestations, these evidence-based regimens have higher effectiveness rates (95%–99%) [18,19] than the regimen approved by the FDA in 2000 (88%–92%) [4,5]. Today, evidence-based regimens are routinely administered throughout the US and the world and are recommended by guidelines of the American College of Obstetricians and Gynecologists, [18] the National Abortion Federation, [20] and the World Health Organization [21].

It is legal and common practice in the US for physicians to prescribe pharmaceuticals off-label; one study estimated that 21% of all US prescriptions are for off-label use [22]. Health care providers prescribing medications off-label have the responsibility to be well informed about the product and to base its use on firm scientific rationale and sound medical evidence [23]. Indeed, it is precisely because of off-label use that abortion providers and researchers have been able to refine the medication abortion regimen to maximize effectiveness and minimize side effects.

Abortion providers in Ohio also used off-label evidence-based regimens for medication abortion. However, in February 2011, an Ohio law took effect mandating that abortion providers use the FDA-approved protocol for medication abortion. The law prohibits off-label use of mifepristone, and thus, at the time it was enacted, it prohibited use of the evidence-based regimens for medication abortion.

In response to the growing body of clinical evidence, the FDA approved a revised label in March 2016 [3,24] to bring the medication abortion protocol in line with the off-label prescribing of mifepristone and misoprostol that had become the standard of care [13,25]. Thus, between February 2011 and March 2016, all abortion providers in the state of Ohio were legally required to use the FDA protocol as approved in 2000.

Other state legislatures passed or enacted similar laws requiring use of the FDA-approved protocol. North Dakota [26] and Texas [27] had such laws in effect. Arizona, Arkansas, and Oklahoma passed similar laws, but they were enjoined by court order. As a result of the FDA decision in March 2016, Ohio’s abortion providers immediately reverted back to the protocol that they were using before 2011 [28]. In the three states where the law is in effect, including Ohio, abortion providers can now legally offer patients medication abortion based on the currently available evidence as long as new research does not further improve clinical best practices.

The primary objective of this study was to examine whether the 2011 Ohio law change from an evidence-based regimen (first column in Table 1) to the FDA regimen (as approved in 2000) was associated with the need for additional intervention following medication abortion. Medication abortions are typically considered effective if no additional interventions, such as subsequent aspiration or repeat dose(s) of misoprostol are required to complete the abortion. Additionally, we sought to examine the number of follow-up visits, continuing pregnancy rate, experience of side effects, proportion of medication abortions (versus other abortion procedures), and average patient charges for medication abortion.

## Materials and Methods

### Data

The University of California, San Francisco (UCSF) Committee on Human Research granted ethical approval for this study (original approval date: 30 June 2014; study number: 14–13766). We compared several medication abortion outcomes and utilization before the law to after the law. Data came from two sources: (1) abstracted patient chart data from four abortion-providing facilities in Ohio and (2) administrative data from the same four facilities.

The UCSF research team provided a full day of on-site training to each of the six data abstractors in the standardized data abstraction protocol, which covered data abstraction methods, basic research principles, ethical conduct of research, and detailed instructions for all data abstraction fields. Each abstractor was given a training manual that they kept on hand as they abstracted data into a standardized electronic form (See S2 Text, “Data Abstraction Protocol”). They abstracted sociodemographic and clinical chart data for all medication abortion patients from 1 y prior to the law’s implementation (January 2010–January 2011) to 3 y post implementation (February 2011–October 2014). Each abstractor received an approximate equal balance of pre- and postlaw charts and was instructed and reminded to enter all data and clinical notes as they appeared in the chart and to use notes fields to explain any errors or discrepancies noticed. Outside of the notes fields, abstractors were instructed not to interpret the data, even if they thought there was an error. Abstractors checked for data entry errors by performing regular checks on charts chosen by the UCSF research team at random. Errors were corrected and addressed by more frequent checks and additional training and clarification. All data were abstracted from paper charts and were entered into and immediately saved on an encrypted and HIPAA-compliant electronic platform that was only accessible to the UCSF research team. For each medication abortion, women typically had the following visits: an information/ultrasound visit, a mifepristone visit, a misoprostol visit (in the postlaw period only), and a follow-up visit. Some patients had additional follow-up visits if needed. To ensure independence among observations, if a patient had more than one medication abortion during the study period, only the first was abstracted. Abstraction occurred between September 2014 and April 2015.

Facility-level administrative data were also collected to assess trends in medication abortions over time. We obtained the total number of abortions and total number of medication abortions from all four sites for each year between 2010 and 2014. We also obtained average patient pricing for medication abortion in 2010 and 2014.

### Measures

Sociodemographic measures abstracted from the patient charts included age, highest level of education, race/ethnicity, insurance status, zip code, height, weight, and previous births. (Insurance status did not necessarily reflect how the patient paid for the abortion.) Clinical information included dates of care, weeks/days gestation, medications administered, patient-reported side effects, diagnoses in the case of adverse events, and additional care following initiation of the medication abortion provided within the facility, or at an outside facility if reported to the abortion facility. We defined the need for additional intervention as needing repeat misoprostol, repeat mifepristone and misoprostol, aspiration, blood transfusion, surgery, or hospital admission following a medication abortion. Adverse event diagnoses included continuing pregnancy, incomplete or possible incomplete abortion, acute hemorrhage, or infection. Distance travelled to abortion care was calculated based on home zip code (the most detailed location information available) to facility using the “traveltime3” STATA module, which utilizes a Google Maps application programming interface (API) to calculate driving distance. Because gestation is not always recorded at the mifepristone visit if it was recorded at the information visit, we imputed gestation at the mifepristone visit based on the number of days since the information visit for 11% of abortions. Body mass index (BMI) was calculated based on height and weight. Days to first follow-up was calculated based on the mifepristone administration date and the first follow-up visit date. When missing, the number of previous births was computed based on number of previous vaginal births, number of previous cesarean sections (c-sections), number of previous pregnancies, number of previous abortions, and number of previous miscarriages. All other data were analyzed as they appeared in the chart.

### Data Analysis

We performed the analysis in four steps. First, we described the characteristics of the study population in the pre- and postlaw periods and compared distributions using chi-squared tests.

Second, we estimated the rate of additional intervention in the pre- and postlaw periods for women who had a medication abortion ≤49 d since their last menstrual period (LMP). To check whether the increases in additional intervention were due to pre-existing time trends, we conducted an interrupted time series analysis. We produced monthly averages of additional intervention rates and constructed a segmented linear regression model including only month, pre/postlaw time period, and a month by pre/postlaw time period interaction to examine trends in rates pre- versus postlaw.

Multivariable logistic regression was used to model the adjusted odds of requiring an additional intervention, controlling for potential confounders and utilizing robust standard error estimation. Because only one abortion per woman was included, there was no need to account for within-woman clustering for multiple abortions. We adjusted for factors that were both available in the charts and that have been suggested or demonstrated in the literature as potentially affecting risk of an unsuccessful medication abortion [17,29,30]. The multivariable models included the following covariates: age, highest level of education, race/ethnicity, insurance status, distance travelled, BMI, gestation at mifepristone administration visit, number of previous births, and site. An abortion was the unit of analysis. All variables in the models were categorical, and many included a “not in chart” category that was retained in the models.

To account for within-clinic similarities and trends, as well as to take into account existing time trends at the site level, we constructed univariate and multivariable mixed-effects autoregressive models with the clinic-month as the unit of analysis, as recommended by a peer reviewer. The rate of additional intervention was calculated for each clinic in each month, and all covariates in the models above were aggregated to the clinic-month level (e.g., % of abortions at clinic 1 in month 1 with private insurance). An autoregressive residual structure was included to account for correlation within sites over time; a range of plausible autoregressive orders were tested. Because of a relatively small number of clusters, we used restricted maximum likelihood and Kenward-Roger denominator degrees-of-freedom adjustment [31].

We conducted the following set of post hoc sensitivity analyses to test the robustness of the finding of increased need for additional intervention in the postlaw period, all of which were recommended by peer reviewers.

1. We replicated the adjusted model for additional intervention with only those cases for which we had complete data for all factors in the model and excluded the “not in chart” category to assess any potential changes in statistical significance.
2. We replicated the adjusted model excluding those women who did not return for a follow-up visit to determine whether the outcomes were influenced by follow-up rates.
3. We conducted a post hoc analysis to test the hypothesis that the lengthened recommended time to follow-up (5–14 d following misoprostol administration prelaw, lengthened to 14 d following the misoprostol visit postlaw) may have increased the additional time “at risk,” thereby driving up intervention rates. For these analyses, we excluded the 28% of the sample who did not return for a follow-up visit. We first conducted a t-test to compare average days to first follow-up visit between pre- and postlaw charts, to assess whether days to follow-up actually increased. We then conducted univariate and multivariable Poisson regression analysis for ungrouped data [32] using person-date level data with a log-time offset to determine whether the association between pre/post-law and additional intervention was sensitive to days to follow-up.
4. We explored how the exclusion of second and higher order abortions may have impacted our results by examining data from one clinic site where higher order abortions were inadvertently abstracted but subsequently excluded from the analytic sample. We compared intervention rates among those with only one abortion to those with second and higher order abortions.
5. Finally, to understand the extent of the impact of missing charts on the results, we developed counterfactuals using extreme assumptions about the intervention rate among those missing charts and whether they were from the pre- or postlaw periods and then calculated pre- and postintervention rates based on the two scenarios. We started with an assumption that all missing charts were from the prelaw period and that, in this group, the intervention rate was the upper confidence limit from the postlaw period. Then, we assumed all missing charts were in the post-law period and that, in this group, the intervention rate was the lower confidence limit from the prelaw period. We then calculated overall intervention rates.

Third, we calculated frequencies of adverse events diagnosed, number of follow-up visits, and patient-reported side effects among the total sample. We tabulated adverse events for all women who had an intervention, reported at any follow-up visit, by time period. We also constructed two multivariable models examining the factors associated with no follow-up visits and 2+ follow-up visits. Because the postlaw protocol requires an additional visit for misoprostol administration, it is possible that this provides more opportunity for women to report side effects in the postlaw period. To assess this bias, we calculated side effects in two ways: we estimated the percent of women reporting at least one side effect at any visit and the percent of women reporting at least one side effect at any visit excluding the misoprostol administration visit. As with intervention rates, we conducted a sensitivity analysis as recommended by a peer reviewer, using univariate and multivariable Poisson regression analysis for ungrouped data with a log-time offset to determine whether the association between pre/post-law and reported side effects was impacted by the number of days to follow-up. Multivariable models for both number of follow-up visits and side effects controlled for age, highest level of education, race/ethnicity, insurance status, distance travelled, BMI, gestation at mifepristone administration visit, previous births, and site. All analyses described above were limited to ≤49 d from LMP because medication abortions were not performed after 49 d in the postlaw period and because of the known association between weeks gestation and need for additional intervention [17,19].

Fourth, we used facility-level administrative data to calculate the proportion of abortions that were medication abortions versus other abortion procedures at all facilities as well as the mean charge for patients in US dollars for medication abortion in 2010 and 2014. The mean charge was weighted by the total number of medication abortion patients at each facility in each year. Statistical significance was set at *p* < 0.05. All statistical analyses were conducted using Stata version 13.1.

Our original primary hypothesis was that among women at ≤49 d gestation, the odds of additional intervention would increase after the implementation of the FDA regimen, adjusting for potential confounders (See S1 Text, “Preliminary Analysis Plan”). We planned to use multivariable logistic regression to assess this question and, after examining the data, added the interrupted time series analysis. We also planned to look for change in the sociodemographic characteristics of patients and in the overall use of medication abortion. We also conducted several analyses that we had planned to explore but did not have a priori stated hypotheses. These included pre- versus postlaw comparisons of the prevalence of adverse events diagnosed, side effects, and facility-level mean charges for medication abortion. We did not plan to conduct all of the post hoc sensitivity analyses, but many were suggested by our peer reviewers, and we believed they were important to test the robustness of our results.

## Results

We requested data on all medication abortions in the study period for a total of 5,095 charts: 2,783 prelaw and 2,312 postlaw. Of these, 930 abstracted charts (18%) were excluded because they did not meet inclusion criteria: they were second or higher order abortions for the same patient during the study period, misidentified patients (aspiration rather than medication abortions), treatment for early pregnancy loss/miscarriage, and cases in which the woman did not have an abortion at that facility.

Additionally, 352 charts were missing (6.9%), most often because the chart was transferred to an off-site warehouse or another health center when a patient received care for a separate reason and the chart subsequently could not be located. We could not determine whether missing charts were from pre- or postlaw periods. An additional 17 charts were dropped from the analysis because they had either entire pages or key dates missing. Among the 3,796 available charts, we included 73% (*n* = 2,783, 1,156 prelaw and 1,627 postlaw) in this analysis because they were ≤49 d from LMP. Most abortions excluded from the prelaw period were excluded because they were >49 d from LMP (medication abortion could not be performed at these gestations in the postlaw period), resulting in a higher number of excluded abortions in the prelaw period than in the postlaw period. We restricted our analyses to ≤49 d from LMP to make the pre- and postlaw samples as comparable as possible given the known association between gestational age and medication abortion effectiveness and outcomes. This sample size afforded us statistical power of 87% to detect a difference of three percentage points or greater in abortion intervention rates between the pre- and postlaw periods, based on an expected rate of 5.2% in the prelaw period [29].

The characteristics of the sample population are listed in Table 2. Over one-third (34%) of the sample were ages 20–24, one-fourth (25%) were ages 25–29, and another one-fourth (25%) were ages 30–39. Most women had a high school diploma or equivalent (37%) or some college (29%). The majority of the women were white (70%). Almost one-third (31%) of the women had private health insurance, and 17% had Medicaid/Medicare. Another 27% did not have health insurance. Most women (86%) travelled <50 mi for abortion care, although 13% travelled 50 mi or more. Half of the women (50%) were healthy weight, and the majority (59%) were at gestations of 42–49 d (6–7 wk). The largest proportion of the women (52%) had not previously given birth. There were significant differences between the prelaw and postlaw populations in this sample by education, race, insurance status, gestation, and number of previous births. The pre- and postlaw populations did not differ significantly by age, distance travelled, BMI, or site visited.

**Table 2:** Characteristics of the pre- and postlaw populations ≤49 d from LMP at four Ohio abortion-providing facilities, 2010–2014.

	Total	Prelaw	Postlaw	*P*-value
*n*, #	2,783	1,156	1,627	
Age, # (%)				0.318
<20 y	360 (12.9)	165 (14.3)	195 (12.0)	
20–24 y	945 (34.0)	385 (33.3)	560 (34.4)	
25–29 y	697 (25.0)	274 (23.7)	422 (26.0)	
30–39 y	684 (24.6)	291 (25.2)	393 (24.2)	
40+ y	97 (3.5)	41 (3.5)	56 (3.4)	
Highest Level of Education, # (%)				<0.001
Less than high school	240 (8.6)	117 (10.1)	123 (7.6)	
High school diploma or GED	1,021 (36.7)	443 (38.3)	578 (35.5)	
Associate’s degree/some college	798 (28.7)	320 (27.7)	478 (29.4)	
Bachelor’s degree or higher	550 (19.8)	172 (14.9)	378 (23.2)	
Not in chart	174 (6.3)	104 (9.0)	79 (4.3)	
Race/Ethnicity, # (%)				0.010
White	1,948 (70.0)	788 (68.2)	1,160 (71.3)	
Black	495 (17.8)	239 (20.7)	256 (15.7)	
Latina	112 (4.0)	38 (3.3)	74 (4.5)	
Asian/Pacific Islander	108 (3.9)	44 (3.8)	64 (3.9)	
Other/Not in chart	120 (4.3)	47 (4.1)	73 (4.5)	
Insurance, # (%)				<0.001
Private	856 (30.8)	308 (26.6)	548 (33.7)	
Medicaid/Medicare	477 (17.1)	199 (17.2)	278 (17.1)	
None	745 (26.8)	348 (30.1)	397 (24.4)	
Not in chart	705 (25.3)	301 (26.0)	404 (24.8)	
Distance Travelled, # (%)				0.413
<50 mi	2,397 (86.1)	1,002 (86.7)	1,395 (85.7)	
50+ mi	358 (12.9)	140 (12.1)	218 (13.4)	
Not in chart	28 (1.0)	14 (1.2)	14 (0.9)	
Body Mass Index (BMI), # (%)				0.188
Underweight (<18.5)	98 (3.5)	39 (3.4)	59 (3.6)	
Healthy weight (18.5–25)	1,388 (49.9)	549 (47.5)	839 (51.6)	
Overweight (25–30)	682 (24.5)	299 (25.9)	383 (23.5)	
Obese (30–35)	248 (8.9)	100 (8.7)	148 (9.1)	
Morbidly obese (35+)	206 (7.4)	92 (8.0)	114 (7.0)	
Not in chart	161 (5.8)	77 (6.7)	84 (5.2)	
Gestation at Mifepristone Visit, # (%)				<0.001
Up to 34 d from LMP (up to 5 wk)	274 (9.8)	155 (13.4)	119 (7.3)	
35–41 d from LMP (5–6 wk)	872 (31.3)	398 (34.4)	474 (29.2)	
42–49 d from LMP (6–7 wk)	1,637 (58.8)	603 (52.2)	1,034 (63.6)	
Number of Previous Births, # (%)				0.031
0	1,459 (52.4)	569 (49.2)	890 (54.7)	
1	634 (22.8)	272 (23.5)	362 (22.2)	
2	434 (15.6)	205 (17.7)	229 (14.1)	
3+	244 (8.8)	104 (9.0)	140 (8.6)	
Not in chart	12 (0.4)	6 (0.5)	6 (0.4)	
Site, # (%)				0.085
1	1,707 (61.3)	719 (62.2)	988 (60.7)	
2	191 (6.9)	71 (6.1)	120 (7.4)	
3	134 (4.8)	67 (5.8)	67 (4.1)	
4	751 (27.0)	299 (25.9)	452 (27.8)	

GED, general educational development test.

### Need for Additional Intervention

**Fig 1:**
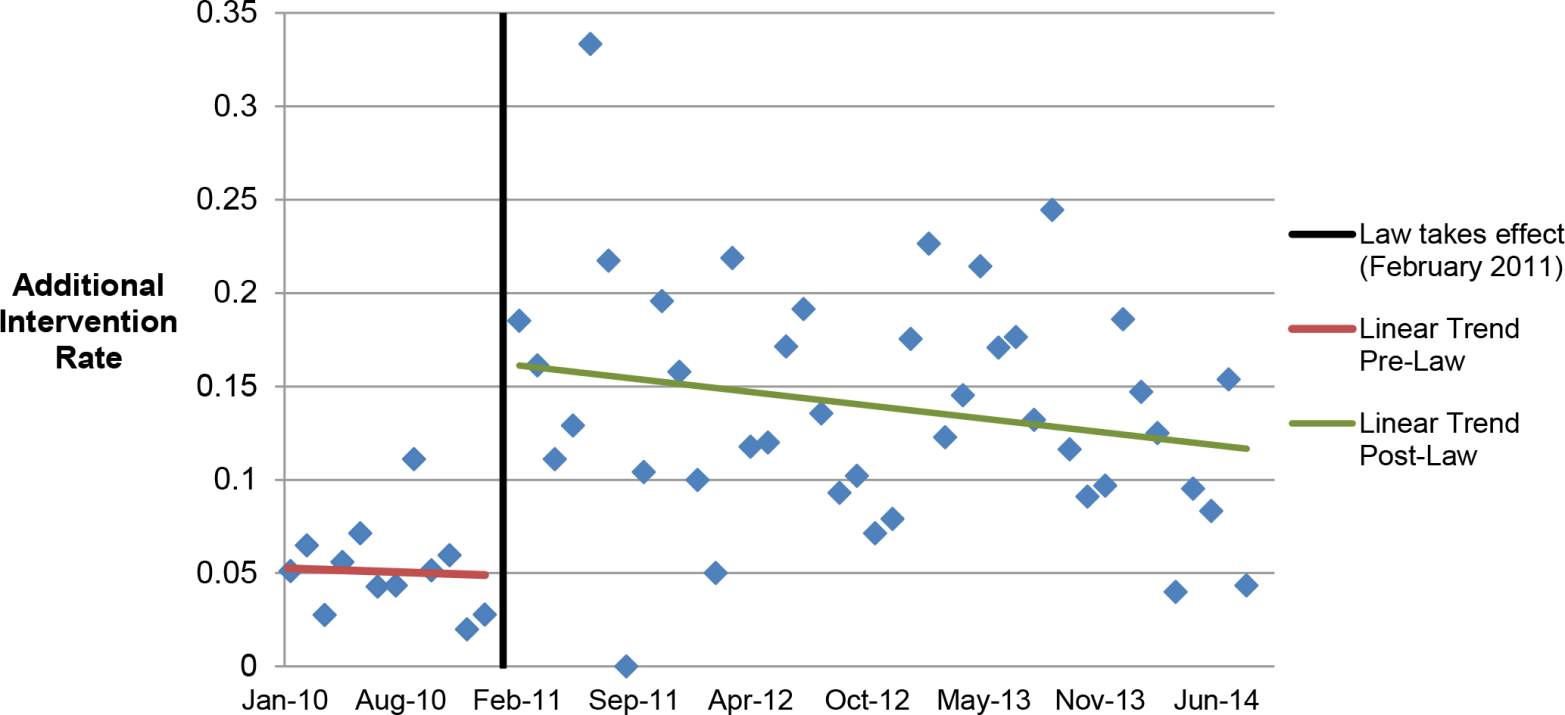
Average additional intervention rate by month. Linear trends in additional intervention rates in the months pre- and postlaw.

Among the 2,783 medication abortion patients ≤49 d gestation, 4.9% (95% CI: 3.7%–6.2%) (57/1,156) in the prelaw and 14.3% (95% CI: 12.6%–16.0%) (233/1,627) in the postlaw period required an additional intervention (*p* < 0.001). To better understand the intervention rates by time before or since the law’s initiation, we produced monthly averages of additional intervention rates and examined linear trends in rates prelaw versus postlaw (Fig 1). The results of the segmented regression analysis find a significant step change in the intervention rate of 12.4% (*p* = 0.001) between pre- and postlaw periods and no evidence of a change in slope (*p* = 0.74) from the pre- to postlaw period.

In the multivariable model, women who had medication abortions in the postlaw period had three times the odds of requiring at least one additional intervention as women in the prelaw period (adjusted odds ratio [AOR] = 3.11, 95% CI: 2.27–4.27) (Table 3).

**Table 3:** Multivariable model of characteristics associated with additional interventions following medication abortions up to 49 d (*n* = 2,783).

	Adjusted OR	*P*-value	95% CI
Time Period			
Prelaw	Ref	Ref	Ref
Postlaw	3.11	<0.001	2.27–4.27
Age			
<20 y	1.20	0.401	0.78–1.84
20–24 y	Ref	Ref	Ref
25–29 y	1.28	0.150	0.91–1.79
30–39 y	1.30	0.191	0.88–1.91
40+ y	1.16	0.702	0.55–2.46
Highest Level of Education			
Less than high school diploma	0.69	0.181	0.39–1.19
High school diploma or GED	Ref	Ref	Ref
Associate’s degree/some college	0.76	0.077	0.55–1.03
Bachelor’s degree or higher	0.67	0.034	0.46–0.97
Not in chart	0.70	0.252	0.38–1.29
Race/Ethnicity			
White	Ref	Ref	Ref
Black	1.37	0.077	0.97–1.94
Latina	0.78	0.478	0.38–1.57
Asian/Pacific Islander	1.11	0.761	0.57–2.16
Other/Not in chart	1.35	0.323	0.74–2.44
Insurance Status			
Private	Ref	Ref	Ref
Medicaid/Medicare	0.48	0.002	0.30–0.77
None	0.85	0.368	0.60–1.21
Not in chart	0.79	0.243	0.53–1.18
Distance Travelled			
<50 mi	Ref	Ref	Ref
50+ mi	1.17	0.397	0.81–1.68
Not in chart	0.69	0.618	0.16–2.98
Body Mass Index (BMI)			
Underweight (<18.5)	0.98	0.960	0.49–1.98
Healthy weight (18.5–25)	Ref	Ref	Ref
Overweight (25–30)	1.09	0.559	0.81–1.47
Obese (30–35)	1.00	0.983	0.62–1.59
Morbidly obese (35+)	0.74	0.279	0.43–1.30
Not in chart	0.58	0.501	0.12–2.80
Gestation at Mifepristone Visit			
Up to 34 d from LMP (up to 5 wk)	Ref	Ref	Ref
35–41 d from LMP (5–6 wk)	1.55	0.139	0.87–2.77
42–49 d from LMP (6–7 wk)	2.22	0.005	1.27–3.87
Number of Previous Births			
0	Ref	Ref	Ref
1	0.92	0.651	0.64–1.32
2	0.96	0.846	0.63–1.47
3+	0.94	0.817	0.54–1.63
Not in chart	1.76	0.540	0.29–10.85
Site			
1	Ref	Ref	Ref
2	0.80	0.422	0.47–1.38
3	0.74	0.731	0.13–4.26
4	0.93	0.712	0.43–1.36

OR, odds ratio; Ref, reference group.

Adjusting for time period (pre- versus postlaw), other characteristics had significant associations with additional interventions (Table 3). Women with Medicaid were less likely to have additional interventions than women with private insurance (AOR = 0.48, 95% CI: 0.30–0.77). Women with a bachelor’s degree or higher levels of education were less likely to require additional intervention (AOR = 0.67, 95% CI: 0.46–0.97). The likelihood of requiring additional intervention increased with gestational age regardless of the study period: women having abortions at 42–49 d (6–7 wk) gestation were significantly more likely to require additional intervention than women <35 d (<5 wk) gestation (AOR = 2.22, 95% CI: 1.27–3.87). Age, race/ethnicity, distance travelled, BMI, previous births, and site were not found to be associated with additional interventions.

The clinic-month level mixed effects autoregressive analyses found a statistically significant increase in intervention rate following the law in both the univariate analysis (absolute increase of 11.7%, 95% CI: 2.6%–20.8%) and multivariable analysis (absolute increase of 9.4%, 95% CI: 4.0%–18.4%) (Table 4).

**Table 4:** Mixed effects models of clinic-month intervention rates with autoregressive residuals (*n* = 179).

	Coefficient	*P*-value	95% CI	Coefficient	*P*-value	95% CI
Time Period						
Prelaw	Ref	Ref	Ref	Ref	Ref	Ref
Postlaw	0.117	0.012	0.026–0.208	0.094	0.041	0.004 to 0.184
Month	−0.001	0.235	−0.004–0.001	−0.0004	0.758	−0.003 to 0.002
Age						
<20 y	-	-	-	0.082	0.503	−0.159 to 0.322
20–24 y	-	-	-	Ref	Ref	Ref
25–29 y	-	-	-	−0.010	0.939	−0.257 to 0.238
30–39 y	-	-	-	−0.204	0.162	−0.491 to 0.083
40+ y	-	-	-	−0.126	0.461	−0.465 to 0.212
Highest Level of Education						
Less than high school diploma	-	-	-	−0.087	0.464	−0.321 to 0.147
High school diploma or GED	-	-	-	Ref	Ref	Ref
Associate’s degree/some college	-	-	-	−0.247	0.049	−0.493 to −0.001
Bachelor’s degree or higher	-	-	-	−0.127	0.353	−0.397 to 0.142
Not in chart	-	-	-	−0.392	0.031	−0.747 to −0.036
Race/Ethnicity						
White	-	-	-	Ref	Ref	Ref
Black	-	-	-	0.171	0.056	−0.004 to 0.346
Latina	-	-	-	0.132	0.358	−0.151 to 0.415
Asian/Pacific Islander	-	-	-	0.013	0.913	−0.216 to 0.241
Other/Not in chart	-	-	-	0.064	0.543	−0.143 to 0.271
Insurance Status						
Private	-	-	-	Ref	Ref	Ref
Medicaid/Medicare	-	-	-	−0.330	0.014	−0.587 to −0.073
None	-	-	-	−0.097	0.356	−0.316 to 0.122
Not in chart	-	-	-	−0.143	0.047	−0.284 to −0.002
Distance Travelled						
<50 mi	-	-	-	Ref	Ref	Ref
50+ mi	-	-	-	−0.132	0.082	−0.282 to 0.017
Not in chart	-	-	-	0.151	0.731	−0.714 to 1.016
Body Mass Index (BMI)						
Underweight (<18.5)	-	-	-	0.001	0.997	−0.367 to 0.369
Healthy weight (18.5–25)	-	-	-	Ref	Ref	Ref
Overweight (25–30)	-	-	-	0.066	0.707	−0.281 to 0.414
Obese (30–35)	-	-	-	0.011	0.957	−0.404 to 0.426
Morbidly obese (35+)	-	-	-	−0.122	0.574	−0.551 to 0.307
Not in chart	-	-	-	−0.086	0.626	−0.4235 to 0.263
Gestation at Mifepristone Visit						
Up to 34 d from LMP (up to 5 wk)	-	-	-	Ref	Ref	Ref
35–41 d from LMP (5–6 wk)	-	-	-	0.054	0.755	−0.288 to 0.395
42–49 d from LMP (6–7 wk)	-	-	-	0.044	0.797	−0.296 to 0.384
Number of Previous Births						
0	-	-	-	Ref	Ref	Ref
1	-	-	-	0.345	<0.001	0.172 to 0.518
2	-	-	-	0.264	0.021	0.041 to 0.486
3+	-	-	-	0.327	<0.001	0.148 to 0.506
Not in chart	-	-	-	−0.146	0.737	−1.003 to 0.712

### Sensitivity Analyses

The series of post hoc sensitivity analyses confirmed the significantly higher rate of intervention in the postlaw period, compared to the prelaw period. When we reran the adjusted model using only those patients with complete data for every factor in the model (e.g., excluding any abortions with a “not in chart” value, *n* included = 1,801), the indicator variable for prelaw versus postlaw remained significantly associated with additional intervention (AOR = 5.06, *p* ≤ 0.001, 95% CI: 3.24–7.88), as did bachelor’s degree or higher education, gestation, and insurance type (S1 Table).

We also reran the adjusted model including only patients who had at least one follow-up visit (72% of the sample, *n* = 833 prelaw and *n* = 1,172 postlaw). In this model, the indicator variable for pre- versus postlaw remained significantly associated with additional intervention (AOR = 3.12, *p* ≤ 0.001, 95% CI: 2.24–4.34). Bachelor’s degree or higher education, insurance, and gestation also remained significantly associated with additional intervention. In this model, travelling 50 mi or more to the clinic was also associated with additional interventions (versus travelling less than 50 mi) (S2 Table).

Results from an analysis of whether additional time “at risk” was driving increased intervention rates found that, indeed, average time from mifepristone visit to first follow-up was significantly longer in the postlaw period (16.0 d prelaw versus 17.9 d postlaw, *p* < 0.001). However, in univariate and multivariable Poisson models taking into account days to follow-up with a log-time offset, women in the postlaw period remained significantly more likely to require an additional intervention (incidence rate ratio [IRR] = 2.33, *p* < 0.001, 95% CI: 1.72–3.14; adjusted IRR [aIRR] = 2.17, *p* < 0.001, 95% CI: 1.56–3.02) (S3 Table). Thus, additional time at risk (additional time to follow-up) was not associated with increased need for additional intervention.

Analysis of data from the one facility where all medication abortions were abstracted demonstrated that 3.9% of all women had more than one abortion during the study period. At that facility, 7.5% of first abortions during the study period required an additional intervention, and 8.9% of second and higher order abortions required additional interventions, a difference that was not statistically significant.

To test whether the 352 missing charts influenced the results, we estimated the intervention rates under two extreme scenarios. First, we assumed that all missing charts were in the prelaw period and that, in this group, the intervention rate was the upper confidence limit from the postlaw period (16.0%). If this were the case, intervention rates would have remained significantly lower in the prelaw period than in the postlaw period (7.6% versus 14.3%, *p* < 0.001). Second, we assumed all missing charts were in the postlaw period and that, in this group, the intervention was the lower confidence limit from the prelaw period (3.7%). If this were the case, intervention again would have remained significantly lower in the prelaw period than in the postlaw period (4.9% versus 12.4%, *p* < 0.001).

### Interventions and Adverse Event Diagnoses

**Fig 2:**
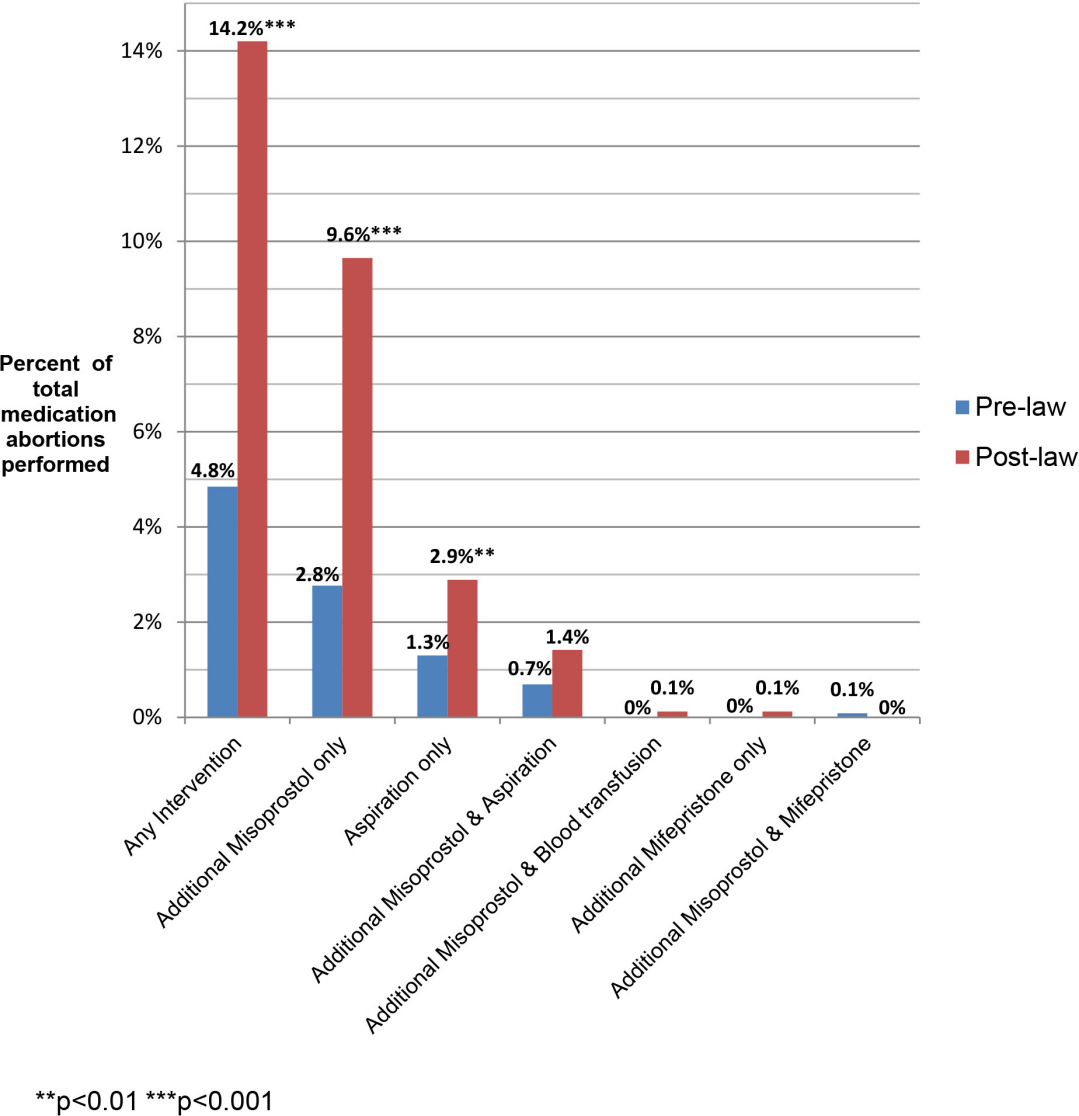
Additional interventions following medication abortion, by type of intervention.

The most common subsequent interventions in both periods were an additional misoprostol dose (8.0%) or aspiration (3.3%). Two patients had repeat doses of mifepristone because they vomited the first dose, one patient had repeat doses of both mifepristone and misoprostol, and two patients had blood transfusions (Fig 2).

The majority of women in both pre- and postlaw periods had no adverse events recorded in their charts (96% overall, 98% prelaw, 95% postlaw, *p* < 0.001). The continuing pregnancy rate among all abortions was 0.3% (*n* = 9) and not statistically different pre- and postlaw (0.1% prelaw, 0.5% postlaw, *p* = 0.06). The rate of incomplete or possible incomplete abortion was 2.4% and was higher postlaw (1.1% prelaw, 3.2% postlaw, *p* < 0.001). An additional 7% of all women had no adverse event recorded but received a subsequent intervention (3.2% prelaw, 9.0% postlaw, *p* < 0.001).

### Follow-up Visits

A similar proportion of women in pre- and postlaw periods did not return for a follow-up visit (27.9% prelaw, 28.0% postlaw, *p* = 0.99). Several demographic factors were associated with not returning for a follow-up visit; in both time periods, women travelling 50 or more mi to the clinic, women using Medicaid as insurance, and women having one or more previous births were associated with greater likelihood of not returning for follow-up compared to women travelling less than 50 mi, women paying out of pocket, and women with no previous births. Women aged 30–39 y and Asian women were also more likely to return for follow-up than women aged 20–24 y and white women. Pre/post-law time period was not significantly associated with returning for follow-up (S4 Table).

Additional interventions were reflected in follow-up visits; the percentage of women requiring two or more follow-up visits increased from 4.2% (95% CI: 3.0%–5.3%) in the prelaw period to 6.8% (95% CI: 5.5%–8.0%) in the postlaw period (*p* = 0.003). In an adjusted model, the difference remained statistically significant (AOR 1.80, 95% CI: 1.22–2.65) (S4 Table). Overall, 1.7% of all women visited an emergency department after their abortion for abortion-related care; this did not differ significantly pre- and postlaw (1.2% prelaw, 2.0% postlaw, *p* = 0.10).

### Side Effects

Overall, 12.6% of women reported at least one side effect during their medication abortion: 8.4% in the prelaw period and 15.6% in the postlaw period (*p* < 0.001). After adjustment for potential confounders, the difference remains significant (AOR 2.01, 95% CI: 1.55–2.60) (S5 Table). The most common reported side effects were nausea and/or vomiting (7.4% of women). Fewer than 1% of women reported fever, chills, dizziness/lightheadedness, diarrhea, or vasovagal fainting, and 5.4% reported any “other” side effect, including pain, swelling, fatigue, vaginal discharge, and headache. The primary source of the pre/post-law difference was nausea and/or vomiting: 4.5% reported nausea and/or vomiting in the prelaw period, compared to 9.5% in the postlaw period (*p* < 0.001). When we excluded those who reported side effects at the misoprostol administration visit in the postlaw period, we found that the difference between pre- and postlaw rates of reporting any side effect remained significant (8.4% versus 11.5%, *p* = 0.008). This significance remains in the adjusted model (AOR 1.42, 95% CI: −1.08–1.86) (S5 Table). As with intervention rates, we also examined whether “time at risk” was associated with reporting of any side effect; in univariate and multivariable Poisson models accounting for days to follow-up, pre/post-law remained significantly associated with reported side effects (IRR = 1.45, *p* = 0.003, 95% CI: 1.13–1.85, aIRR 1.46, *p* = 0.005, 95% CI: 1.22–2.65) (S6 Table).

### Trends in Medication Abortion

**Fig 3:**
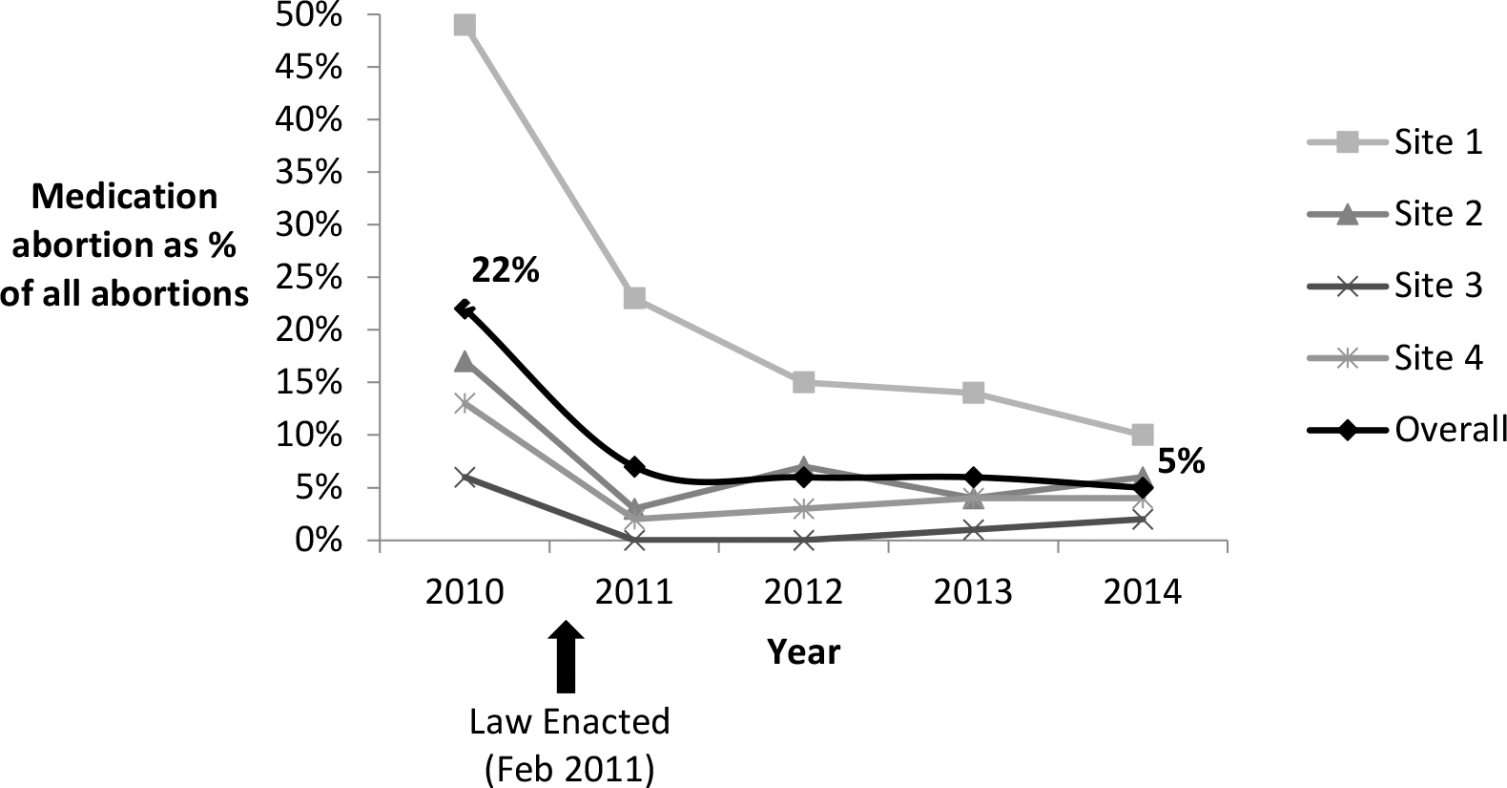
Proportion of abortions that were medication abortions at four Ohio facilities, 2010–2014.

Based on facility-level administrative data on all medication abortions at the participating facilities (≤63 d from LMP in the prelaw period and ≤49 d from LMP in the postlaw period), there was an overall decline in the proportion of abortions that were medication abortions (Fig 3). In 2010, before the law went into effect, 22% (95% CI: 20.8%–22.3%) of all abortions were medication abortions. After the law, this proportion declined to 7% (95% CI: 6.8%–7.8%) in 2011, 6% (95% CI: 5.4%–6.2%) in 2012, 6% (95% CI: 5.1%–6.0%) in 2013, and 5% (95% CI: 4.8%–5.6%) in 2014 (2010 versus 2014, *p* < 0.001). One facility stopped providing medication abortions after implementation of the law through January 2013.

The average patient charge for medication abortion rose from US$426 to US$551 between 2010 and 2014, representing a 29% increase in nominal dollars and a 16% increase after adjusting for inflation in medical prices [33].

## Discussion

A 2011 Ohio law was enacted that required the use of the FDA-approved regimen for medication abortion rather than the evidence-based regimen supported by several international guidelines but yet to be approved by the FDA. This study finds that while the provision of medication abortion was still safe and effective in Ohio, the 2011 law change was associated with greater need for additional intervention, more visits, more side effects, and higher costs for women who have medication abortions.

Additionally, we observed a trend of declining use of medication abortion, which is consistent with other analyses of state-level data from Ohio [34] and with experiences in Texas after a similar law was passed there [35]. This decline is opposite to national trends, where medication abortion is increasing as a proportion of all abortions [36,37].

The law increased the logistical burdens on women who have medication abortions. Between February 2011 and March 2016, women needed to make a minimum of four visits instead of two, resulting in potential increases in transportation and childcare costs and more time away from work and school [38]. In recent years, the number of facilities in Ohio has decreased from 14 to 8 [39], and some providers had stopped providing medication abortion as a result of this law [40]. Thus, many women who prefer this abortion method needed to travel further to obtain it. More than 10% of the patients in our sample travelled over 50 mi to get to the abortion provider; for women having to make four visits, this adds up to more than 400 mi travelled. The requirement of misoprostol administration in the provider’s office also led to cases of vomiting and other side effects beginning on the journey home.

The increased rate of side effects in the postlaw period may be due to the higher dose of mifepristone and the oral route of misoprostol administration. Previous studies report higher rates of nausea and vomiting [4,41] and more severe bleeding [4] with 600 mg versus 200 mg of mifepristone. While the misoprostol dose in the postlaw period was lower, clinical trials have demonstrated that oral administration is associated with higher rates of gastrointestinal effects [42], nausea, vomiting, and diarrhea [43,44] than vaginal administration.

That women with Medicaid coverage were less likely to have a subsequent intervention than women with private insurance is likely explained by differences in socioeconomic status and the ability to pay for additional health care. Because Ohio’s Medicaid program generally does not cover abortion, patients with Medicaid pay for their abortion out of pocket. It is possible such patients are reluctant to return to the abortion provider despite unusual symptoms, because of fear of financial costs of additional follow-up care. Alternatively, women with Medicaid may have been more likely to seek care from emergency departments (EDs) or other sources of care. Indeed data show that Medicaid recipients are more likely to use EDs than privately insured populations [45,46].

We found significant changes in the characteristics of women able to obtain medication abortions before and after the law. In particular, a larger proportion of women in the postlaw period were having abortions at 42–49 d. We hypothesize that more women had abortions at 42–49 d in the postlaw period because they were approaching the gestational limit (49 d) to have a medication abortion, whereas women at 42–49 d in the prelaw period did not experience the same urgency. It is also possible that accommodating additional required visits for all medication abortion patients resulted in longer clinic wait times for next available appointments, but we did not collect data on this. At the same time, we found greater proportions of women with characteristics that one would expect to be associated with lower intervention rates in the postlaw period, including higher education, white race, private insurance, and fewer previous births. However, this study did not include women obtaining medication abortions greater than 49 d in the prelaw period. Future studies should investigate how the medication abortion law may have impacted the sociodemographic characteristics of women who were able to access medication abortions.

There may be alternative explanations for the increased rates of interventions and side effects found in this study after the law change. Various components of the Affordable Care Act that increased the number of women with public and private insurance were enacted between 2010 and 2014 [47]. However, most provisions that expanded care did not go into effect until 2013 and 2014 [48,49], and health plans offered in Ohio’s health exchange under the Affordable Care Act (ACA) can only cover abortion in cases when the woman's life is endangered or in cases of rape or incest [50], decreasing the likelihood that the ACA contributed to the change we saw in the data in 2011. Another explanation is that implementation of the FDA-approved protocol required an additional visit to report side effects, leading to higher rates of side effects. However, our analysis found that after removing those reported at the additional visit, the rate of side effects in the postlaw period was still higher. The law also required a greater number of days (14 d) between mifepristone and follow-up visits than the evidence-based protocol (5–14 d), allowing for more time for symptoms to develop that may need intervention. Our analyses checked for this and found that the odds of intervention or side effects were not sensitive to days to follow-up. Additionally, providers in the postlaw period, knowing that they were using an older protocol, may have been inclined to resort to intervention more frequently. Nevertheless, even if these explanations were true, the resulting conclusion would still be that something about implementation of the law and/or the FDA-approved protocol was associated with higher rates of intervention.

This study has several limitations. First, it was a pre/post-observational study with no control group that was not exposed to the law. However, the outcomes we examined (additional interventions, continuing pregnancy, and side effects) were directly related to the route of administration and dose of medications used for abortion. Additionally, the medication abortion regimen was changed in response to the law, and the trend data indicated that the outcomes changed when the law took effect in 2011. Thus, it is unlikely that the changes we found after the law’s implementation were due to factors unrelated to the law. Second, with many patients not returning for a follow-up visit, some women may have experienced signs or symptoms of problems but did not return to report them to the original abortion facility. It is possible that some women may have gone to an ED in the case of an adverse event without informing the abortion provider. If that type of visit was reported to the original abortion provider and documented in her chart, we included those visits; however, there is no reason to believe that such loss to follow-up would occur at higher rates in the prelaw than the postlaw period. In a sensitivity analysis in which we excluded those with no follow-up visit, the magnitude and significance of the difference in pre- versus postlaw intervention rates remained the same. Third, despite the great lengths the abstractors went through to locate charts at nearby facilities, there were still missing charts, and we had no information about whether they were from the pre- or postlaw period. We have no reason to believe that missingness was associated with whether or not the patient had an intervention, but older charts (prelaw) may have been more likely to be missing given that they were sent to off-site storage or another facility and had more time to become lost. Results from the sensitivity analyses testing the most extreme assumptions about these missing charts demonstrate that they had little impact on our conclusions that intervention rates were significantly higher in the postlaw period. Fourth, second and higher order abortions were excluded from the study. We were able to quantify the frequency of such abortions at only one of the four facilities (where higher order abortions were abstracted but were excluded) and found that 3.9% of women (*n* = 56) had more than one abortion. When we compared intervention rates between first abortions and second and higher order abortions in a sensitivity analysis, we found no significant differences; thus, the impact of this exclusion is likely minimal. Finally, the data came from the abstraction of medical records, which allows for the potential for abstractor bias. There was no practical way to blind the abstractors regarding the pre- or postlaw period given that the two protocols were so different and the dates of visits were in the charts. However, we were conscious of abstractor bias, and each abstractor underwent rigorous training that aimed to minimize bias.

This study also has several strengths. It assessed changes associated with implementation of a new law, comparing rates of subsequent intervention during two periods of time across multiple facilities, with a sufficiently large sample size. Trend data support that change occurred as the law went into effect. Second, we used data on only one abortion per woman, which ensures independence of all observations in the analysis. Finally, it is unique in evaluating the impact of legislation using objective clinical outcomes.

Even though the original FDA-approved protocol was recently updated to bring it in line with current evidence, best practices will continue to be updated, and laws like Ohio’s prohibit clinicians from practicing medicine based on the latest developments in clinical research. For example, in March 2016 the FDA deemed there was insufficient evidence for allowing providers to call in prescriptions for mifepristone to pharmacies. Clinical trials that test the safety of pharmacy access are currently underway [51,52]. Similarly, the FDA approved an increase in use among women 49 to 70 d from LMP, but clinical trials are now testing safety and efficacy of medication abortion regimens within the first 77 d from LMP [53]. This law will continue to require physicians to provide care that may fall below the accepted standard of care, placing them in an ethical dilemma.

### Conclusions

These findings demonstrate that enactment of an Ohio abortion law restricting the use of medication abortion was associated with significant increases in medical interventions, side effects, and costs. The law required physicians to alter their evidence-based practices to provide care that may have been more burdensome for their patients than before the law was implemented [25]. The law was also associated with a decrease in the proportion of abortions that are medication abortions. There is no evidence that the change in law led to improved abortion outcomes. Indeed, our findings suggest the opposite.

## Supporting Information

S1 STROBE Checklist STROBE checklist. (DOCX)

S1 Table Multivariable model of characteristics associated with additional interventions following medication abortions up to 49 d, complete data only. (DOCX)

S2 Table Multivariable model of characteristics associated with additional interventions following medication abortions up to 49 d, those who returned for follow-up only. (DOCX)

S3 Table Poisson models of intervention rates with log days to follow-up offset (includes those who returned for follow-up only). (DOCX)

S4 Table Odds of returning for two or more follow-up visits, adjusted models. (DOCX)

S5 Table Odds of reporting at least one side effect, adjusted models. (DOCX)

S6 Table Poisson models of reported side effect rates with log days to follow-up offset (includes those who returned for follow-up only). (DOCX)

S1 Text Preliminary analysis plan. (PDF)

S2 Text Data abstraction protocol. (PDF)

## Abbreviations

ACA: Affordable Care Act
aIRR: adjusted incidence rate ratio
AOR: adjusted odds ratio
API: application programming interface
BMI: body mass index
c-section: cesarean section
ED: emergency department
FDA: United States Food and Drug Administration
GED: general educational development test
IRR: incidence rate ratio
LMP: last menstrual period
UCSF: University of California, San Francisco
