# Supplementary material for: Comparison of Outcomes before and after Ohio's Law Mandating Use of the FDA-Approved Protocol for Medication Abortion: A Retrospective Cohort Study
Source: PLoS Med. 2016 Aug 30;13(8):e1002110. doi: 10.1371/journal.pmed.1002110 (PMC5004901; doi:10.1371/journal.pmed.1002110)
Supplement: S1 STROBE Checklist — (DOCX) [file pmed.1002110.s001.docx]

STROBE Statement—checklist of items that should be included in reports of observational studies

**Ohio's Law Mandating Use of the FDA-Approved Protocol for Medication Abortion: A Comparison of Outcomes Before and After the Law in a Retrospective Cohort** Ushma D Upadhyay, Nicole E Johns, Sarah L Combellick, Julia E Kohn, Lisa M Keder, Sarah CM Roberts

|  | | Item No | Recommendation |
| --- | --- | --- | --- |
| **Title and abstract** | | 1 | (*a*) Indicate the study’s design with a commonly used term in the title or the abstract  **Abstract: “We used a retrospective cohort design”** |
|  |  |  | (*b*) Provide in the abstract an informative and balanced summary of what was done and what was found  **“We used a retrospective cohort design, comparing outcomes of medication abortion patients in the pre-law period to those in the post-law period. Sociodemographic and clinical chart data were abstracted from all medication abortion patients from one year prior to the law’s implementation (January 2010–January 2011) to three years post-implementation (February 2011–October 2014) at four abortion-providing healthcare facilities in Ohio. Outcome data were analyzed for all women undergoing abortion at ≤49 days gestation during the study period. The main outcomes were: need for additional intervention following medication abortion (such as aspiration, repeat misoprostol, and blood transfusion), frequency of continuing pregnancy, reports of side effects, and the proportion of abortions that were medication abortions (vs. other abortion procedures).**  **Among the 2,783 medication abortions ≤49 days gestation, 4.9% (95% CI: 3.7–6.2%) in the pre-law and 14.3% (95% CI: 12.6–16.0%) in the post-law period required one or more additional interventions. Women obtaining a medication abortion in the post-law period had 3 times the odds of requiring an additional intervention as women in the pre-law period (AOR=3.11, 95% CI: 2.27–4.27). In a mixed effects multivariable model that uses facility-months as the unit of analysis to account for lack of independence by site, we found that the law change was associated with an 9.4% (95% CI: 4.0%-18.4%) absolute increase in the rate of requiring an additional intervention. The most common subsequent intervention in both periods was an additional misoprostol dose and was most commonly administered to treat incomplete abortion. The percentage of women requiring two or more follow-up visits increased from 4.2% (95% CI: 3.0%-5.3%) in the pre-law period to 6.2% (95% CI: 5.5%-8.0%) in the post-law period (p=0.003). Continuing pregnancy was rare (0.3%). Overall, 12.6% of women reported at least one side effect during their medication abortion: 8.4% (95% CI: 6.8%-10.0%) in the pre-law period and 15.6% (95% CI: 13.8%-17.3%) in the post-law period (p<0.001). Medication abortions fell from 22% (95% CI: 20.8%-22.3%) of all abortions the year before the law went into effect (2010) to 5% (95%CI: 4.8%-5.6%) three years after (2014) (p<0.001). Average patient charge increased from $426 in 2010 to $551 in 2014, representing a 16% increase after adjusting for inflation in medical prices. The primary limitation to the study is that it was a pre/post observational study with no control group that was not exposed to the law.”** |
| Introduction | | | |
| Background/rationale | | 2 | Explain the scientific background and rationale for the investigation being reported  **First 8 paragraphs of the Introduction.** |
| Objectives | | 3 | State specific objectives, including any prespecified hypotheses  **“The primary objective of this study was to examine whether the 2011 Ohio law change from an evidence-based regimen (first column in Table 1) to the FDA regimen (as approved in 2000) was associated with the need for additional intervention following medication abortion.”** |
| Methods | | | |
| Study design | | 4 | Present key elements of study design early in the paper  **First paragraph of the Methods: “We compared several medication abortion outcomes and utilization before the law to after the law.”** |
| Setting | | 5 | Describe the setting, locations, and relevant dates, including periods of recruitment, exposure, follow-up, and data collection  **“Data came from two sources: 1) Abstracted patient chart data from four abortion-providing facilities in Ohio, and 2) administrative data from the same four facilities. …**  **“The UCSF research team provided a full day of on-site training to each of the six data abstractors in the standardized data abstraction protocol which covered data abstraction methods, basic research principles, ethical conduct of research, and detailed instructions for all data abstraction fields. Each abstractor was given a training manual that they kept on hand as they abstracted data into a standardized electronic form (See S2 Text. Data Abstraction Protocol). They abstracted sociodemographic and clinical chart data for all medication abortion patients from one year prior to the law’s implementation (January 2010–January 2011) to three years post-implementation (February 2011–October 2014). Each abstractor received an approximate equal balance of pre- and post-law charts and were instructed and reminded to enter all data and clinical notes as they appeared in the chart and to use notes fields to explain any errors or discrepancies noticed. Outside of the notes fields, abstractors were instructed not to interpret the data, even if they thought there was an error. Abstractors checked for data entry errors by performing regular checks on charts chosen by the UCSF research team at random. Errors were corrected and addressed by more frequent checks and additional training and clarification. All data were abstracted from paper charts and were entered into and immediately saved on an encrypted and HIPAA-compliant electronic platform that was only accessible to the UCSF research team. For each medication abortion, women typically had the following visits: an information/ultrasound visit, a mifepristone visit, a misoprostol visit (in the post-law period only), and a follow-up visit. Some patients had additional follow-up visits if needed. To ensure independence among observations, if a patient had more than one medication abortion during the study period, only the first was abstracted. Abstraction occurred between September 2014 and April 2015.”**  **“Facility-level administrative data were also collected to assess trends in medication abortions over time. We obtained the total number of abortions and total number of medication abortions from all four sites for each year between 2010 and 2014. We also obtained average patient pricing for medication abortion in 2010 and 2014.”** |
| Participants | | 6 | (*a*) *Cohort study*—Give the eligibility criteria, and the sources and methods of selection of participants. Describe methods of follow-up.  **“They abstracted sociodemographic and clinical chart data for all medication abortion patients from one year prior to the law’s implementation (January 2010–January 2011) to three years post-implementation (February 2011–October 2014).**  **For each medication abortion, women typically had the following visits: an information/ultrasound visit, a mifepristone visit, a misoprostol visit (in the post-law period only), and a follow-up visit. Some patients had additional follow-up visits if needed. To ensure independence among observations, if a patient had more than one medication abortion during the study period, only the first was abstracted.”**  *Case-control study*—Give the eligibility criteria, and the sources and methods of case ascertainment and control selection. Give the rationale for the choice of cases and controls  *Cross-sectional study*—Give the eligibility criteria, and the sources and methods of selection of participants |
|  |  |  | (*b*) *Cohort study*—For matched studies, give matching criteria and number of exposed and unexposed  *Case-control study*—For matched studies, give matching criteria and the number of controls per case |
| Variables | | 7 | Clearly define all outcomes, exposures, predictors, potential confounders, and effect modifiers. Give diagnostic criteria, if applicable  **Paragraph under section heading, “Measures”.** |
| Data sources/ measurement | | 8* | For each variable of interest, give sources of data and details of methods of assessment (measurement). Describe comparability of assessment methods if there is more than one group  **Paragraph under section heading, “Measures”.** |
| Bias | | 9 | Describe any efforts to address potential sources of bias  **“The UCSF research team provided a full day of on-site training to each of the six data abstractors in the standardized data abstraction protocol which covered data abstraction methods, basic research principles, ethical conduct of research, and detailed instructions for all data abstraction fields. Each abstractor was given a training manual that they kept on hand as they abstracted data into a standardized electronic form (See S2 Text. Data Abstraction Protocol).”**  **“Each abstractor received an approximate equal balance of pre- and post-law charts and were instructed and reminded to enter all data and clinical notes as they appeared in the chart and to use notes fields to explain any errors or discrepancies noticed. Outside of the notes fields, abstractors were instructed not to interpret the data, even if they thought there was an error. Abstractors checked for data entry errors by performing regular checks on charts chosen by the UCSF research team at random. Errors were corrected and addressed by more frequent checks and additional training and clarification. All data were abstracted from paper charts and were entered into and immediately saved on an encrypted and HIPAA-compliant electronic platform that was only accessible to the UCSF research team.”** |
| Study size | | 10 | Explain how the study size was arrived at.  **Methods and Results sections:**  **Methods: They abstracted sociodemographic and clinical chart data for all medication abortion patients from one year prior to the law’s implementation (January 2010–January 2011) to three years post-implementation (February 2011–October 2014).**  **Results: Among the 3,796 available charts, we included 73% (n=2,783, 1,156 pre-law and 1,627 post-law) in this analysis because they were ≤49 days LMP. …** **This sample size afforded us statistical power of 87% to detect a difference of three percentage points or greater in abortion intervention rates between the pre- and post-law periods, based on an expected rate of 5.2% in the pre-law period [26].** |
| Quantitative variables | | 11 | Explain how quantitative variables were handled in the analyses. If applicable, describe which groupings were chosen and why  **Paragraphs in the Data Analysis section.** |
| Statistical methods | | 12 | (*a*) Describe all statistical methods, including those used to control for confounding  **Paragraphs in the Data Analysis section.** |
|  |  |  | (*b*) Describe any methods used to examine subgroups and interactions  **No interaction terms have been tested.** |
|  |  |  | (*c*) Explain how missing data were addressed  **“All variables in the model were categorical and many included a “not in chart” category which was retained in the model. As a post-hoc sensitivity analysis, we replicated the adjusted model for additional intervention with only those cases for which we had complete data for all factors in the model, and excluding the “not in chart” category to assess any potential changes in statistical significance.”** |
|  |  |  | (*d*) *Cohort study*—If applicable, explain how loss to follow-up was addressed  **Within the methods section: “B) In another sensitivity test, we replicated the adjusted model excluding those women who did not return for a follow-up visit to determine whether the outcomes were influenced by follow up rates.”**  *Case-control study*—If applicable, explain how matching of cases and controls was addressed  *Cross-sectional study*—If applicable, describe analytical methods taking account of sampling strategy |
|  |  |  | (*e*) Describe any sensitivity analyses  **Data analysis. “We conducted the following set of post-hoc sensitivity analyses to test the robustness of the finding of increased need for additional intervention in the post-law period, all of which were recommended by peer reviewers. A) We replicated the adjusted model for additional intervention with only those cases for which we had complete data for all factors in the model, and excluding the “not in chart” category to assess any potential changes in statistical significance. B) In another sensitivity test, we replicated the adjusted model excluding those women who did not return for a follow-up visit to determine whether the outcomes were influenced by follow up rates. C) We also conducted a post-hoc analysis to test the hypothesis that the lengthened recommended time to follow-up (5-14 days following misoprostol administration pre-law, lengthened to 14 days following misoprostol visit post-law) may have increased the additional time “at risk,” thereby driving up intervention rates. For these analyses we excluded 28% of the sample who did not return for a follow-up visit. We first conducted a t-test to compare average days to first follow-up visit between pre- and post-law charts, to assess whether days to follow-up actually increased. We then conducted univariate and multivariable Poisson regression analysis for ungrouped data [32] using person-date level data with a log-time offset to determine whether the association between pre/post law and additional intervention was sensitive to days to follow-up. D) We also explored how the exclusion of second and higher order abortions may have impacted our results by examining data from one clinic site where higher order abortions were inadvertently abstracted but subsequently excluded from the analytic sample. We compared intervention rates among those with only one abortion to those with second and higher order abortions. E) Finally, to understand the extent of the impact of missing charts on the results, we developed counterfactuals using extreme assumptions about the intervention rate among those missing charts and whether they were from the pre- or post-law periods and then calculated pre- and post- intervention rates based on the two scenarios. We started with an assumption that all missing charts were from the pre-law period and that in this group, the intervention rate was the upper confidence limit from the post-law period. Then, we assumed all missing charts were in the post-law period and that in this group, the intervention rate was the lower confidence limit from the pre-law period. We then calculated overall intervention rates.”** |
| Results | | | |
| Participants | 13* | (a) Report numbers of individuals at each stage of study—eg numbers potentially eligible, examined for eligibility, confirmed eligible, included in the study, completing follow-up, and analysed  **First two paragraphs of Results section.** | |
|  |  | (b) Give reasons for non-participation at each stage  **First two paragraphs of Results section.** | |
|  |  | (c) Consider use of a flow diagram.  **We have decided that the participant sample is easy enough to follow without a diagram.** | |
| Descriptive data | 14* | (a) Give characteristics of study participants (eg demographic, clinical, social) and information on exposures and potential confounders  **Table 1 and third paragraph in the Results section. “The characteristics of the sample population are listed in Table 2. Over one-third (34%) of the sample were ages 20-24, one-fourth (25%) were ages 25-29 and another one-fourth (25%) were ages 30-39. Most women had a high school diploma or equivalent (37%) or some college (29%). The majority of women were white (70%). Almost one-third (31%) of women had private insurance, and 17% had Medicaid/Medicare. Another 27% did not have health insurance. Most women (86%) travelled <50 miles for abortion care, although 13% travelled 50 miles or more. Half of women (50%) were healthy weight and the majority (59%) were at gestations of 42-49 days (6-7 weeks). The largest proportion of women (52%) had not previously given birth. There were significant differences between the pre-law and post-law populations in this sample by education, race, insurance status, gestation, and number of previous births. The pre- and post-law populations did not differ significantly by age, distance travelled, BMI, or site visited.”** | |
|  |  | (b) Indicate number of participants with missing data for each variable of interest  **Table 1 (Not in chart category)** | |
|  |  | (c) *Cohort study*—Summarise follow-up time (eg, average and total amount)  **From results section:**  **“Results from an analysis of whether additional time “at risk” was driving increased intervention rates, found that indeed, average time from mifepristone visit to first follow-up was significantly longer in the post law period (16.0 days pre-law vs 17.9 days post-law, p<0.001).”** | |
| Outcome data | 15* | *Cohort study*—Report numbers of outcome events or summary measures over time  **First paragraph under section heading, “Need for Additional Intervention”**  **“Among the 2,783 medication abortion patients ≤49 days gestation, 4.9% (95% CI: 3.7–6.2%) (57/1156) in the pre-law and 14.3% (95% CI: 12.6–16.0%) (233/1627) in the post-law period required an additional intervention (p<0.001).”** | |
|  |  | *Case-control study—*Report numbers in each exposure category, or summary measures of exposure | |
|  |  | *Cross-sectional study—*Report numbers of outcome events or summary measures | |
| Main results | 16 | (*a*) Give unadjusted estimates and, if applicable, confounder-adjusted estimates and their precision (eg, 95% confidence interval). Make clear which confounders were adjusted for and why they were included.  Unadjusted:  “**Among the 2,783 medication abortion patients ≤49 days gestation, 4.9% (57/1156) in the pre-law and 14.3% (233/1627) in the post-law period required an additional intervention (p<0.001).”**  **Adjusted:**  **“In the multivariable model, women who had medication abortions in the post-law period had three times the odds of requiring at least one additional intervention as women in the pre-law period (AOR=3.11, 95% CI: 2.27–4.27) (Table 3).”** | |
|  |  | (*b*) Report category boundaries when continuous variables were categorized  **N/A** | |
|  |  | (*c*) If relevant, consider translating estimates of relative risk into absolute risk for a meaningful time period  **N/A** | |
| Other analyses | 17 | Report other analyses done—eg analyses of subgroups and interactions, and sensitivity analyses  **Additional paragraphs in Results section.** | |
| Discussion | | | |
| Key results | 18 | Summarise key results with reference to study objectives  **First paragraph of Discussion section:**  **“A 2011 Ohio law was enacted which required the use of the FDA-approved regimen for medication abortion rather than the evidence based regimen supported by several international guidelines but yet to be approved by the FDA. This study finds that while the provision of medication abortion was still safe and effective in Ohio, the 2011 law change was associated with greater need for additional intervention, more visits, more side effects, and higher costs for women who have medication abortions.”** | |
| Limitations | 19 | Discuss limitations of the study, taking into account sources of potential bias or imprecision. Discuss both direction and magnitude of any potential bias  **Limitations paragraph in Discussion section.** | |
| Interpretation | 20 | Give a cautious overall interpretation of results considering objectives, limitations, multiplicity of analyses, results from similar studies, and other relevant evidence  **Conclusion (last paragraph).** | |
| Generalisability | 21 | Discuss the generalisability (external validity) of the study results  **Last paragraph before Conclusions.** | |
| Other information | | | |
| Funding | 22 | Give the source of funding and the role of the funders for the present study and, if applicable, for the original study on which the present article is based.  **Provided.** | |

*Give information separately for cases and controls in case-control studies and, if applicable, for exposed and unexposed groups in cohort and cross-sectional studies.

**Note:** An Explanation and Elaboration article discusses each checklist item and gives methodological background and published examples of transparent reporting. The STROBE checklist is best used in conjunction with this article (freely available on the Web sites of PLoS Medicine at http://www.plosmedicine.org/, Annals of Internal Medicine at http://www.annals.org/, and Epidemiology at http://www.epidem.com/). Information on the STROBE Initiative is available at www.strobe-statement.org.
