# Supplementary material for: Comparison of Outcomes before and after Ohio's Law Mandating Use of the FDA-Approved Protocol for Medication Abortion: A Retrospective Cohort Study
Source: PLoS Med. 2016 Aug 30;13(8):e1002110. doi: 10.1371/journal.pmed.1002110 (PMC5004901; doi:10.1371/journal.pmed.1002110)
Supplement: S1 Table — (DOCX) [file pmed.1002110.s002.docx]

S1 Table. Multivariable model of characteristics associated with additional interventions following medication abortions up to 49 days, complete data only (N=1801)

|  | Adjusted OR | P-Value | 95% CI |
| --- | --- | --- | --- |
| Time period |  |  |  |
| Pre-law | Ref | Ref | Ref |
| Post-law | 5.06 | <0.001 | 3.24–7.88 |
| Age |  |  |  |
| <20 | 1.24 | 0.403 | 0.75–2.06 |
| 20**–**24 | Ref |  | Ref |
| 25**–**29 | 1.60 | 0.025 | 1.06–2.42 |
| 30**–**39 | 1.44 | 0.155 | 0.87–2.39 |
| 40+ | 0.95 | 0.920 | 0.37–2.46 |
| Highest level of education |  |  |  |
| Less than high school diploma | 0.62 | 0.143 | 0.33–1.18 |
| High school diploma or GED | Ref |  | Ref |
| Associates degree/some college | 0.63 | 0.015 | 0.43–0.91 |
| Bachelors degree or higher | 0.42 | <0.001 | 0.27–0.68 |
| Race/Ethnicity |  |  |  |
| White | Ref | Ref | Ref |
| Black | 1.91 | 0.003 | 1.24–2.95 |
| Latina | 1.02 | 0.965 | 0.47–2.20 |
| Asian/Pacific Islander | 1.35 | 0.477 | 0.59–3.10 |
| Other | 1.01 | 0.982 | 0.36–2.88 |
| Insurance Status |  |  |  |
| Private | Ref | Ref | Ref |
| Medicaid/Medicare | 0.49 | 0.008 | 0.29–0.83 |
| None | 0.86 | 0.448 | 0.58–1.27 |
| Distance Travelled |  |  |  |
| <50 miles | Ref | Ref | Ref |
| 50+ miles | 1.10 | 0.671 | 0.70–1.74 |
| Body Mass Index (BMI) |  |  |  |
| Underweight (<18.5) | 1.38 | 0.415 | 0.64–2.99 |
| Healthy weight (18.5-25) | Ref | Ref | Ref |
| Overweight (25-30) | 1.12 | 0.526 | 0.78–1.62 |
| Obese (30-35) | 0.93 | 0.819 | 0.51–1.69 |
| Morbidly obese (35+) | 0.56 | 0.122 | 0.27–1.17 |
| Gestation at mifepristone visit |  |  |  |
| Up to 34 days LMP (up to 5 weeks) | Ref | Ref | Ref |
| 35–41 days LMP (5–6 weeks) | 1.85 | 0.079 | 0.93–3.67 |
| 42–49 days LMP (6–7 weeks) | 2.53 | 0.006 | 1.31–4.88 |
| Number of previous births |  |  |  |
| 0 | Ref | Ref | Ref |
| 1 | 0.82 | 0.396 | 0.52–1.30 |
| 2 | 0.78 | 0.375 | 0.45–1.36 |
| 3+ | 0.49 | 0.078 | 0.23–1.08 |
| Site |  |  |  |
| 1 | Ref | Ref | Ref |
| 2 | 1.1 | 0.776 | 0.57–2.15 |
| 3 | - | - | - |
| 4 | 0.85 | 0.522 | 0.51–1.41 |
