# Supplementary material for: Comparison of Outcomes before and after Ohio's Law Mandating Use of the FDA-Approved Protocol for Medication Abortion: A Retrospective Cohort Study
Source: PLoS Med. 2016 Aug 30;13(8):e1002110. doi: 10.1371/journal.pmed.1002110 (PMC5004901; doi:10.1371/journal.pmed.1002110)
Supplement: S2 Table — (DOCX) [file pmed.1002110.s003.docx]

S2 Table. Multivariable model of characteristics associated with additional interventions following medication abortions up to 49 days, those who returned for follow-up only (N=2005)

|  | Adjusted OR | P-value | 95% CI |
| --- | --- | --- | --- |
| Time period |  |  |  |
| Pre-law | Ref | Ref | Ref |
| Post-law | 3.12 | <0.001 | 2.24–4.34 |
| Age |  |  |  |
| <20 | 1.30 | 0.293 | 0.80–2.10 |
| 20**–**24 | Ref | Ref | Ref |
| 25**–**29 | 1.35 | 0.101 | 0.94–1.94 |
| 30**–**39 | 1.15 | 0.525 | 0.75–1.76 |
| 40+ | 1.15 | 0.725 | 0.52–2.54 |
| Highest level of education |  |  |  |
| Less than high school diploma | 0.72 | 0.281 | 0.39–1.31 |
| High school diploma or GED | Ref | Ref | Ref |
| Associates degree/some college | 0.77 | 0.134 | 0.55–1.08 |
| Bachelors degree or higher | 0.62 | 0.024 | 0.41–0.94 |
| Not in chart | 0.68 | 0.261 | 0.35–1.33 |
| Race/Ethnicity |  |  |  |
| White | Ref | Ref | Ref |
| Black | 1.35 | 0.121 | 0.92–1.96 |
| Latina | 0.77 | 0.506 | 0.36–1.65 |
| Asian/Pacific Islander | 0.85 | 0.670 | 0.41–1.78 |
| Other/Not in chart | 1.46 | 0.235 | 0.78–2.75 |
| Insurance Status |  |  |  |
| Private | Ref | Ref | Ref |
| Medicaid/Medicare | 0.45 | 0.002 | 0.27–0.75 |
| None | 0.76 | 0.148 | 0.52–1.10 |
| Not in chart | 0.76 | 0.198 | 0.50–1.16 |
| Distance Travelled |  |  |  |
| <50 miles | Ref | Ref | Ref |
| 50+ miles | 1.60 | 0.020 | 1.07–2.37 |
| Not in chart | 0.76 | 0.720 | 0.16–3.48 |
| Body Mass Index (BMI) |  |  |  |
| Underweight (<18.5) | 0.81 | 0.582 | 0.39–1.70 |
| Healthy weight (18.5-25) | Ref | Ref | Ref |
| Overweight (25-30) | 1.15 | 0.387 | 0.84–1.58 |
| Obese (30-35) | 1.05 | 0.865 | 0.62–1.76 |
| Morbidly obese (35+) | 0.71 | 0.250 | 0.40–1.27 |
| Not in chart | 0.69 | 0.661 | 0.13–3.58 |
| Gestation at mifepristone visit |  |  |  |
| Up to 34 days LMP (up to 5 weeks) | Ref | Ref | Ref |
| 35–41 days LMP (5–6 weeks) | 1.26 | 0.453 | 0.68–2.33 |
| 42–49 days LMP (6–7 weeks) | 1.99 | 0.021 | 1.11–3.58 |
| Number of previous births |  |  |  |
| 0 | Ref | Ref | Ref |
| 1 | 1.15 | 0.469 | 0.79–1.69 |
| 2 | 1.24 | 0.357 | 0.78–1.97 |
| 3+ | 1.41 | 0.247 | 0.79–2.54 |
| Not in chart | 2.29 | 0.471 | 0.24–21.91 |
| Site |  |  |  |
| 1 | Ref | Ref | Ref |
| 2 | 0.70 | 0.218 | 0.39–1.24 |
| 3 | 0.55 | 0.526 | 0.09–3.43 |
| 4 | 0.89 | 0.556 | 0.59–1.32 |
