# Supplementary material for: Comparison of Outcomes before and after Ohio's Law Mandating Use of the FDA-Approved Protocol for Medication Abortion: A Retrospective Cohort Study
Source: PLoS Med. 2016 Aug 30;13(8):e1002110. doi: 10.1371/journal.pmed.1002110 (PMC5004901; doi:10.1371/journal.pmed.1002110)
Supplement: S3 Table — (DOCX) [file pmed.1002110.s004.docx]

S3 Table. Poisson models of intervention rates with log days to follow-up offset (includes those who returned for follow-up only) (N=31,815 person-days)

|  | IRR | P-value | 95% CI | aIRR | P-value | 95% CI |
| --- | --- | --- | --- | --- | --- | --- |
| Time period |  |  |  |  |  |  |
| Pre-law | Ref | Ref | Ref | Ref | Ref | Ref |
| Post-law | 2.33 | <0.001 | 1.72–3.14 | 2.17 | <0.001 | 1.56–3.02 |
| Age |  |  |  |  |  |  |
| <20 | - |  | - | 1.19 | 0.448 | 0.76–1.87 |
| 20**–**24 | - |  | - | Ref | Ref | Ref |
| 25**–**29 | - |  | - | 1.31 | 0.109 | 0.94–1.82 |
| 30**–**39 | - |  | - | 1.16 | 0.445 | 0.79–1.70 |
| 40+ | - |  | - | 1.38 | 0.375 | 0.68–2.84 |
| Highest level of education |  |  |  |  |  |  |
| Less than high school diploma | - |  | - | 0.76 | 0.356 | 0.43–1.36 |
| High school diploma or GED | - |  | - | Ref | Ref | Ref |
| Associates degree/some college | - |  | - | 0.81 | 0.192 | 0.60–1.11 |
| Bachelors degree or higher | - |  | - | 0.79 | 0.224 | 0.54–1.16 |
| Not in chart | - |  | - | 0.89 | 0.706 | 0.47–1.66 |
| Race/Ethnicity |  |  |  |  |  |  |
| White | - |  | - | Ref | Ref | Ref |
| Black | - |  | - | 1.15 | 0.439 | 0.81–1.64 |
| Latina | - |  | - | 0.94 | 0.868 | 0.46–1.94 |
| Asian/Pacific Islander | - |  | - | 0.79 | 0.513 | 0.40–1.58 |
| Other/Not in chart | - |  | - | 1.09 | 0.754 | 0.63–1.90 |
| Insurance Status |  |  |  |  |  |  |
| Private | - |  | - | Ref | Ref | Ref |
| Medicaid/Medicare | - |  | - | 0.47 | 0.003 | 0.29–0.77 |
| None | - |  | - | 0.86 | 0.405 | 0.60–1.23 |
| Not in chart | - |  | - | 0.77 | 0.161 | 0.54–1.11 |
| Distance Travelled |  |  |  |  |  |  |
| <50 miles | - |  | - | Ref | Ref | Ref |
| 50+ miles | - |  | - | 1.53 | 0.019 | 1.07–2.18 |
| Not in chart | - |  | - | 0.71 | 0.637 | 0.17–2.96 |
| Body Mass Index (BMI) |  |  |  |  |  |  |
| Underweight (<18.5) | - |  | - | 0.85 | 0.630 | 0.43–1.67 |
| Healthy weight (18.5-25) | - |  | - | Ref | Ref | Ref |
| Overweight (25-30) | - |  | - | 1.12 | 0.452 | 0.83–1.51 |
| Obese (30-35) | - |  | - | 1.06 | 0.809 | 0.66–1.70 |
| Morbidly obese (35+) | - |  | - | 0.66 | 0.142 | 0.38–1.15 |
| Not in chart | - |  | - | 0.58 | 0.444 | 0.14–2.37 |
| Gestation at mifepristone visit |  |  |  |  |  |  |
| Up to 34 days LMP (up to 5 weeks) | - |  | - | Ref | Ref | Ref |
| 35–41 days LMP (5–6 weeks) | - |  | - | 1.18 | 0.578 | 0.65–2.15 |
| 42–49 days LMP (6–7 weeks) | - |  | - | 1.64 | 0.089 | 0.93–2.92 |
| Number of previous births |  |  |  |  |  |  |
| 0 | - |  | - | Ref | Ref | Ref |
| 1 | - |  | - | 1.14 | 0.454 | 0.81–1.62 |
| 2 | - |  | - | 1.12 | 0.593 | 0.75–1.67 |
| 3+ | - |  | - | 1.23 | 0.449 | 0.72–2.10 |
| Not in chart | - |  | - | 3.1 | 0.267 | 0.42–22.93 |
| Site |  |  |  |  |  |  |
| 1 | - |  | - | Ref | Ref | Ref |
| 2 | - |  | - | 0.74 | 0.280 | 0.44–1.27 |
| 3 | - |  | - | 0.78 | 0.760 | 0.15–3.96 |
| 4 | - |  | - | 0.90 | 0.562 | 0.65–1.27 |
