# Supplementary material for: Comparison of Outcomes before and after Ohio's Law Mandating Use of the FDA-Approved Protocol for Medication Abortion: A Retrospective Cohort Study
Source: PLoS Med. 2016 Aug 30;13(8):e1002110. doi: 10.1371/journal.pmed.1002110 (PMC5004901; doi:10.1371/journal.pmed.1002110)
Supplement: S4 Table — (DOCX) [file pmed.1002110.s005.docx]

S4 Table. Odds of not returning for follow up visit and for returning for two or more follow-up visits, adjusted models

|  | Not returning for follow-up visit (N=2783) | | | Returning for 2+ follow-up visits (N=2622) | | |
| --- | --- | --- | --- | --- | --- | --- |
|  | Adjusted OR | P-value | 95% CI | Adjusted OR | P-value | 95% CI |
| Time period |  |  |  |  |  |  |
| Pre-law | Ref | Ref | Ref | Ref | Ref | Ref |
| Post-law | 1.03 | 0.732 | 0.86–1.23 | 1.80 | 0.003 | 1.22–2.65 |
| Age |  |  |  |  |  |  |
| <20 | 1.02 | 0.899 | 0.75–1.38 | 1.25 | 0.476 | 0.68–2.29 |
| 20**–**24 | Ref | Ref | Ref | Ref | Ref | Ref |
| 25**–**29 | 0.95 | 0.642 | 0.75–1.19 | 1.32 | 0.216 | 0.85–2.04 |
| 30**–**39 | 0.70 | 0.008 | 0.54–0.91 | 1.23 | 0.412 | 0.75–2.01 |
| 40+ | 0.76 | 0.289 | 0.45–1.27 | 1.51 | 0.381 | 0.60–3.84 |
| Highest level of education |  |  |  |  |  |  |
| Less than high school diploma | 1.06 | 0.739 | 0.76–1.46 | 0.74 | 0.423 | 0.36–1.53 |
| High school diploma or GED | Ref | Ref | Ref | Ref | Ref | Ref |
| Associates degree/some college | 1.02 | 0.849 | 0.82–1.27 | 0.69 | 0.090 | 0.45–1.06 |
| Bachelors degree or higher | 0.87 | 0.307 | 0.66–1.14 | 1.06 | 0.823 | 0.65–1.71 |
| Not in chart | 1.13 | 0.508 | 0.79–1.62 | 0.33 | 0.033 | 0.12–0.92 |
| Race/Ethnicity |  |  |  |  |  |  |
| White | Ref | Ref | Ref | Ref | Ref | Ref |
| Black | 0.87 | 0.232 | 0.68–1.10 | 1.72 | 0.017 | 1.10–2.67 |
| Latina | 0.88 | 0.564 | 0.57–1.36 | 0.83 | 0.687 | 0.32–2.10 |
| Asian/Pacific Islander | 0.41 | 0.003 | 0.23–0.73 | 1.42 | 0.409 | 0.62–3.27 |
| Other/Not in chart | 0.75 | 0.222 | 0.47–1.19 | 1.38 | 0.427 | 0.63–3.03 |
| Insurance Status |  |  |  |  |  |  |
| Private | Ref | Ref | Ref | Ref | Ref | Ref |
| Medicaid/Medicare | 1.27 | 0.083 | 0.97–1.67 | 1.04 | 0.895 | 0.58–1.87 |
| None | 0.96 | 0.736 | 0.76–1.22 | 1.10 | 0.696 | 0.68–1.79 |
| Not in chart | 0.84 | 0.244 | 0.63–1.13 | 1.63 | 0.085 | 0.93–2.85 |
| Distance Travelled |  |  |  |  |  |  |
| <50 miles | Ref | Ref | Ref | Ref | Ref | Ref |
| 50+ miles | 1.86 | <0.001 | 1.46–2.37 | 1.30 | 0.271 | 0.82–2.05 |
| Not in chart | 1.60 | 0.275 | 0.69–3.70 | 0.66 | 0.677 | 0.09–4.71 |
| Body Mass Index (BMI) |  |  |  |  |  |  |
| Underweight (<18.5) | 0.66 | 0.116 | 0.39–1.11 | 1.57 | 0.252 | 0.72–3.42 |
| Healthy weight (18.5-25) | Ref | Ref | Ref | Ref | Ref | Ref |
| Overweight (25-30) | 0.93 | 0.512 | 0.75–1.15 | 0.81 | 0.299 | 0.54–1.21 |
| Obese (30-35) | 1.02 | 0.912 | 0.75–1.38 | 0.78 | 0.424 | 0.42–1.45 |
| Morbidly obese (35+) | 0.85 | 0.335 | 0.60–1.19 | 1.04 | 0.910 | 0.56–1.93 |
| Not in chart | 1.07 | 0.878 | 0.46–2.49 | - | - | - |
| Gestation at mifepristone visit |  |  |  |  |  |  |
| Up to 34 days LMP (up to 5 weeks) | Ref | Ref | Ref | Ref | Ref | Ref |
| 35–41 days LMP (5–6 weeks) | 0.85 | 0.287 | 0.62–1.15 | 1.53 | 0.270 | 0.72–3.23 |
| 42–49 days LMP (6–7 weeks) | 0.92 | 0.585 | 0.69–1.23 | 1.82 | 0.103 | 0.89–3.74 |
| Number of previous births |  |  |  |  |  |  |
| 0 | Ref | Ref | Ref | Ref | Ref | Ref |
| 1 | 1.65 | <0.001 | 1.30–2.09 | 1.13 | 0.613 | 0.70–1.82 |
| 2 | 2.08 | <0.001 | 1.58–2.75 | 1.24 | 0.460 | 0.70–2.19 |
| 3+ | 2.02 | <0.001 | 1.41–2.89 | 1.15 | 0.711 | 0.56–2.37 |
| Not in chart | 2.40 | 0.110 | 0.82–7.05 | 1.59 | 0.679 | 0.18–14.51 |
| Site |  |  |  |  |  |  |
| 1 | Ref | Ref | Ref | Ref | Ref | Ref |
| 2 | 0.87 | 0.483 | 0.60–1.27 | 1.04 | 0.898 | 0.54–2.02 |
| 3 | 0.32 | 0.022 | 0.12–0.85 | - | - | - |
| 4 | 0.87 | 0.315 | 0.66–1.14 | 0.53 | 0.022 | 0.30–0.91 |
