# Supplementary material for: Comparison of Outcomes before and after Ohio's Law Mandating Use of the FDA-Approved Protocol for Medication Abortion: A Retrospective Cohort Study
Source: PLoS Med. 2016 Aug 30;13(8):e1002110. doi: 10.1371/journal.pmed.1002110 (PMC5004901; doi:10.1371/journal.pmed.1002110)
Supplement: S5 Table — (DOCX) [file pmed.1002110.s006.docx]

S5 Table. Odds of reporting at least one side effect, adjusted models

|  | Including misoprostol administration visit (N=2783) | | | Excluding misoprostol administration visit (N=2755) | | |
| --- | --- | --- | --- | --- | --- | --- |
|  | Adjusted OR | P-value | 95% CI | Adjusted OR | P-value | 95% CI |
| Time period |  |  |  |  |  |  |
| Pre-law | Ref | Ref | Ref | Ref | Ref | Ref |
| Post-law | 2.01 | <0.001 | 1.55–2.60 | 1.42 | 0.011 | 1.08–1.86 |
| Age |  |  |  |  |  |  |
| <20 | 1.17 | 0.411 | 0.81–1.68 | 1.08 | 0.687 | 0.73–1.61 |
| 20**–**24 | Ref | Ref | Ref | Ref | Ref | Ref |
| 25**–**29 | 0.84 | 0.293 | 0.62–1.16 | 0.69 | 0.044 | 0.49–0.99 |
| 30**–**39 | 0.84 | 0.332 | 0.59–1.20 | 0.86 | 0.444 | 0.59–1.26 |
| 40+ | 0.96 | 0.920 | 0.48–1.94 | 1.02 | 0.952 | 0.49–2.12 |
| Highest level of education |  |  |  |  |  |  |
| Less than high school diploma | 0.85 | 0.485 | 0.55–1.33 | 0.83 | 0.446 | 0.52–1.34 |
| High school diploma or GED | Ref | Ref | Ref | Ref | Ref | Ref |
| Associates degree/some college | 0.71 | 0.025 | 0.53–0.96 | 0.65 | 0.009 | 0.47–0.90 |
| Bachelors degree or higher | 0.79 | 0.181 | 0.55–1.12 | 0.72 | 0.099 | 0.49–1.06 |
| Not in chart | 0.64 | 0.117 | 0.36–1.12 | 0.58 | 0.078 | 0.31–1.06 |
| Race/Ethnicity |  |  |  |  |  |  |
| White | Ref | Ref | Ref | Ref | Ref | Ref |
| Black | 1.23 | 0.204 | 0.89–1.69 | 1.22 | 0.265 | 0.86–1.72 |
| Latina | 0.83 | 0.542 | 0.45–1.53 | 0.81 | 0.547 | 0.41–1.61 |
| Asian/Pacific Islander | 1.57 | 0.115 | 0.89–2.77 | 1.42 | 0.278 | 0.75–2.67 |
| Other/Not in chart | 1.33 | 0.348 | 0.73–2.43 | 1.48 | 0.220 | 0.79–2.75 |
| Insurance Status |  |  |  |  |  |  |
| Private | Ref | Ref | Ref | Ref | Ref | Ref |
| Medicaid/Medicare | 1.22 | 0.303 | 0.83–1.79 | 1.10 | 0.645 | 0.73–1.67 |
| None | 1.18 | 0.296 | 0.87–1.60 | 1.15 | 0.425 | 0.82–1.60 |
| Not in chart | 1.05 | 0.800 | 0.71–1.56 | 1.07 | 0.761 | 0.70–1.64 |
| Distance Travelled |  |  |  |  |  |  |
| <50 miles | Ref | Ref | Ref | Ref | Ref | Ref |
| 50+ miles | 0.67 | 0.036 | 0.46–0.97 | 0.64 | 0.039 | 0.42–0.98 |
| Not in chart | 0.25 | 0.173 | 0.04–1.82 | - | - | - |
| Body Mass Index (BMI) |  |  |  |  |  |  |
| Underweight (<18.5) | 1.16 | 0.623 | 0.64–2.09 | 1.15 | 0.664 | 0.61–2.20 |
| Healthy weight (18.5-25) | Ref | Ref | Ref | Ref | Ref | Ref |
| Overweight (25-30) | 0.82 | 0.183 | 0.61–1.10 | 0.82 | 0.218 | 0.59–1.13 |
| Obese (30-35) | 1.00 | 0.986 | 0.65–1.52 | 0.98 | 0.946 | 0.62–1.55 |
| Morbidly obese (35+) | 1.24 | 0.349 | 0.79–1.95 | 1.25 | 0.363 | 0.77–2.03 |
| Not in chart | 0.53 | 0.432 | 0.11–2.57 | 0.65 | 0.589 | 0.14–3.05 |
| Gestation at mifepristone visit |  |  |  |  |  |  |
| Up to 34 days LMP (up to 5 weeks) | Ref | Ref | Ref | Ref | Ref | Ref |
| 35–41 days LMP (5–6 weeks) | 1.46 | 0.124 | 0.90–2.37 | 1.52 | 0.117 | 0.90–2.57 |
| 42–49 days LMP (6–7 weeks) | 1.79 | 0.015 | 1.12–2.85 | 1.75 | 0.031 | 1.05–2.91 |
| Number of previous births |  |  |  |  |  |  |
| 0 | Ref | Ref | Ref | Ref | Ref | Ref |
| 1 | 0.75 | 0.100 | 0.54–1.06 | 0.77 | 0.172 | 0.54–1.12 |
| 2 | 0.53 | 0.004 | 0.34–0.82 | 0.61 | 0.041 | 0.38–0.98 |
| 3+ | 0.74 | 0.250 | 0.45–1.23 | 0.81 | 0.435 | 0.47–1.39 |
| Not in chart | 1.17 | 0.854 | 0.22–6.09 | 1.43 | 0.668 | 0.28–7.27 |
| Site |  |  |  |  |  |  |
| 1 | Ref | Ref | Ref | Ref | Ref | Ref |
| 2 | 0.40 | 0.004 | 0.21–0.74 | 0.36 | 0.006 | 0.17–0.74 |
| 3 | 1.31 | 0.753 | 0.25–6.92 | 1.21 | 0.819 | 0.23–6.25 |
| 4 | 0.64 | 0.019 | 0.44–0.93 | 0.67 | 0.047 | 0.45–1.00 |
