# Supplementary material for: Comparison of Outcomes before and after Ohio's Law Mandating Use of the FDA-Approved Protocol for Medication Abortion: A Retrospective Cohort Study
Source: PLoS Med. 2016 Aug 30;13(8):e1002110. doi: 10.1371/journal.pmed.1002110 (PMC5004901; doi:10.1371/journal.pmed.1002110)
Supplement: S6 Table — (DOCX) [file pmed.1002110.s007.docx]

S6 Table. Poisson models of reported side effect rates with log days to follow-up offset (includes those who returned for follow-up only)

|  | N= 31,815 person-days | | | N= 31,543 person-days | | |
| --- | --- | --- | --- | --- | --- | --- |
|  | IRR | P-value | 95% CI | aIRR | P-value | 95% CI |
| Time period |  |  |  |  |  |  |
| Pre-law | Ref | Ref | Ref | Ref | Ref | Ref |
| Post-law | 1.45 | 0.003 | 1.13–1.85 | 1.46 | 0.005 | 1.12–1.91 |
| Age |  |  |  |  |  |  |
| <20 | - |  | - | 1.11 | 0.582 | 0.76–1.63 |
| 20**–**24 | - |  | - | Ref | Ref | Ref |
| 25**–**29 | - |  | - | 0.89 | 0.460 | 0.65–1.22 |
| 30**–**39 | - |  | - | 0.80 | 0.214 | 0.57–1.14 |
| 40+ | - |  | - | 1.11 | 0.755 | 0.56–2.20 |
| Highest level of education |  |  |  |  |  |  |
| Less than high school diploma | - |  | - | 0.73 | 0.182 | 0.45–1.16 |
| High school diploma or GED | - |  | - | Ref | Ref | Ref |
| Associates degree/some college | - |  | - | 0.72 | 0.030 | 0.53–0.97 |
| Bachelors degree or higher | - |  | - | 0.83 | 0.299 | 0.58–1.18 |
| Not in chart | - |  | - | 0.95 | 0.863 | 0.56–1.64 |
| Race/Ethnicity |  |  |  |  |  |  |
| White | - |  | - | Ref | Ref | Ref |
| Black | - |  | - | 1.05 | 0.741 | 0.77–1.45 |
| Latina | - |  | - | 1.13 | 0.678 | 0.63–2.02 |
| Asian/Pacific Islander | - |  | - | 1.30 | 0.355 | 0.74–2.27 |
| Other/Not in chart | - |  | - | 1.12 | 0.693 | 0.64–1.95 |
| Insurance Status |  |  |  |  |  |  |
| Private | - |  | - | Ref | Ref | Ref |
| Medicaid/Medicare | - |  | - | 1.10 | 0.618 | 0.75–1.63 |
| None | - |  | - | 1.11 | 0.506 | 0.81–1.52 |
| Not in chart | - |  | - | 0.91 | 0.593 | 0.63–1.30 |
| Distance Travelled |  |  |  |  |  |  |
| <50 miles | - |  | - | Ref | Ref | Ref |
| 50+ miles | - |  | - | 0.83 | 0.356 | 0.56–1.24 |
| Not in chart | - |  | - | - | - | - |
| Body Mass Index (BMI) |  |  |  |  |  |  |
| Underweight (<18.5) | - |  | - | 1.07 | 0.821 | 0.61–1.88 |
| Healthy weight (18.5-25) | - |  | - | Ref | Ref | Ref |
| Overweight (25-30) | - |  | - | 0.89 | 0.463 | 0.66–1.20 |
| Obese (30-35) | - |  | - | 1.11 | 0.634 | 0.73–1.69 |
| Morbidly obese (35+) | - |  | - | 1.10 | 0.682 | 0.71–1.70 |
| Not in chart | - |  | - | 0.50 | 0.336 | 0.12–2.06 |
| Gestation at mifepristone visit |  |  |  |  |  |  |
| Up to 34 days LMP (up to 5 weeks) | - |  | - | Ref | Ref | Ref |
| 35–41 days LMP (5–6 weeks) | - |  | - | 1.42 | 0.186 | 0.85–2.38 |
| 42–49 days LMP (6–7 weeks) | - |  | - | 1.69 | 0.039 | 1.03–2.80 |
| Number of previous births |  |  |  |  |  |  |
| 0 | - |  | - | Ref | Ref | Ref |
| 1 | - |  | - | 0.85 | 0.327 | 0.61–1.18 |
| 2 | - |  | - | 0.68 | 0.077 | 0.44–1.04 |
| 3+ | - |  | - | 0.86 | 0.568 | 0.52–1.43 |
| Not in chart | - |  | - | 3.64 | 0.072 | 0.89–14.85 |
| Site |  |  |  |  |  |  |
| 1 | - |  | - | Ref | Ref | Ref |
| 2 | - |  | - | 0.47 | 0.015 | 0.26–0.86 |
| 3 | - |  | - | 1.69 | 0.498 | 0.37–7.75 |
| 4 | - |  | - | 0.72 | 0.058 | 0.51–1.01 |
