# Supplementary material for: Comparison of Outcomes before and after Ohio's Law Mandating Use of the FDA-Approved Protocol for Medication Abortion: A Retrospective Cohort Study
Source: PLoS Med. 2016 Aug 30;13(8):e1002110. doi: 10.1371/journal.pmed.1002110 (PMC5004901; doi:10.1371/journal.pmed.1002110)
Supplement: S1 Text — (PDF) [file pmed.1002110.s008.pdf]

Study: Ohio FDA Protocol for Medication Abortion Study

Participating Groups: **UCSF/ANSIRH**
[redacted]

Principal Investigator: Ushma Upadhyay, PhD, MPH, University of California, San Francisco

Co-Investigator(s): Julia Kohn, PhD, MPA, Planned Parenthood Federation of America

Project Consultant/Local University Researcher: Lisa Keder, MD, Ohio State University

Key Contacts:

UCSF ANSIRH: Sarah Combellick, MPH (Project Director)

[redacted] [redacted]

[redacted] [redacted]

[redacted] [redacted]

Primary Study Aim: To assess changes in the need for subsequent intervention after medication abortions, before and after the implementation of the state-required FDA regimen at four Ohio abortion facilities.

Hypotheses:

H1: Among women at ≤ 49 days' gestation, the relative risk of intervention will increase after the implementation of the FDA regimen, adjusting for potential confounders.

Additional exploratory research questions for descriptive analyses:

1. What is the overall effect of the FDA Protocol requirement on demand for medication abortion? How does the profile of medication abortion patients change in terms of sociodemographic characteristics?
2. Is there differential loss to follow up based on zip code? Are women who live further form the facility less likely to return the facility for post-misoprostol follow up visits?

Design:

Observational study with pre-post comparison. Data on all medication abortions performed at selected Ohio health centers for one year before implementation of the law will be compared to data on all medication abortions performed at those same centers following implementation to the present to achieve adequate sample size. Medication abortions from all sites will be pooled. We will compute the intervention rate per 100 abortion procedures before and after implementation and the difference in rates. We will include only procedures ≤ 49 days' gestation.

Total Charts to be Abstracted: We will abstract all Medication abortion charts from all sites 12 months before the law change and 12 months after the law change.

	[redacted]	[redacted]	[redacted]	[redacted]	**Total**
**Pre**	240	1697	200	645	2782
**Post**	220	1561	66	466	2313
**Total**	460	3258	266	1111	5095

Required Data Elements:

- See chart abstraction list
