# Supplementary material for: Comparison of Outcomes before and after Ohio's Law Mandating Use of the FDA-Approved Protocol for Medication Abortion: A Retrospective Cohort Study
Source: PLoS Med. 2016 Aug 30;13(8):e1002110. doi: 10.1371/journal.pmed.1002110 (PMC5004901; doi:10.1371/journal.pmed.1002110)
Supplement: S2 Text — (PDF) [file pmed.1002110.s009.pdf]

Ohio FDA Protocol for Medication Abortion Study

Data Abstraction Manual

Version 1.0
8/18/2014

Table of Contents

I. Study Contact Information 2

II. General Study Information 3

III. Communication Expectations 4

IV. Data Abstraction Overview 4

V. Data Abstraction Instructions 5

VI. Quality Assurance 5

Appendix A: Table of Data Elements with Data Entry Instructions 7

Appendix B: FDA vs. Evidence-Based Protocol for Medication Abortion 27

Appendix C: Visit Flowcharts 28

Appendix D: Data Review Documentation Form 29

Prepared by Sarah Combellick, MPH

I. Study Contact Information

Site Number: __________

Site Name: ____

Data Abstractor Name: _______________________________

Site Supervisor Name: ________________________________

Primary contact person at University of California, San Francisco (UCSF):

Sarah Combellick, Project Director
Office phone: (510) 986-8927
Mobile phone: [redacted]
Email: combellicks@obgyn.ucsf.edu
Office hours: Sarah is available Monday, Tuesday, and Thursday from 11am-7:30pm and Wednesday from 11am-3pm (Eastern Standard Time).

You will have a telephone meeting with Sarah Combellick every

______________________ at ____________________ (Eastern Standard Time).
(day of week) **(time)**

If you need to reach someone at UCSF on a Wednesday afternoon or Friday, or if Sarah is unavailable, contact:
Elise Belusa, Project Coordinator
Office phone: (510) 986-8969
Email: belusae@obgyn.ucsf.edu

Back up contact person if Sarah and Elise are unavailable or if it is outside of UCSF work hours:
Ushma Upadhyay, Principal Investigator
Office phone: (510) 986-8946
Mobile phone: [redacted]
Email: upadhyayu@obgyn.ucsf.edu

II. General Study Information

For this study, we aim to understand the effects of an Ohio law that went into effect in 2011, requiring medication abortions to be provided in accordance to a protocol approved by the U.S. Food and Drug Administration in 2000, compared to the previously used regimen (see **Appendix B** for a table comparing the regimens). We will compare the need for any additional intervention before and after the law was implemented.

This study will use data abstracted from all medical charts of patients receiving medication abortions at four Ohio facilities in both the pre-law and post-law periods: [redacted]. The exact date ranges for chart abstraction vary by site depending on a number of factors. The first day of the post-law period is the date that the site began providing medication abortion with the FDA protocol. The end dates for [redacted] are earlier than the other sites due to the changeover to electronic health records on March 1, 2014.

	[redacted]	[redacted]	[redacted]	[redacted]
**Study timeframe for pre-law Period**	January 1st, 2010 – January 31, 2011			
**Study timeframe for post-law Period**	June 1st, 2011 – February 28, 2014	March 1st, 2011 – February 28, 2014	January 15, 2013 – August 31, 2014	February 23, 2011 – August 31, 2014

Information from a total of approximately 5,000 patient charts will be abstracted. We anticipate that data collection will take place between August and December, 2014. The methods for this study have been approved by the UCSF Committee on Human Research.

III. Communication Expectations

As you begin collecting data, communicate regularly with your site supervisor and the UCSF contact about ANY questions you have about data collection. It is nearly always better to ask than to assume or guess. This helps ensure that data is collected accurately and consistently across study sites. You will never be penalized for asking too many questions.

Please also talk to us about any questions, concerns or suggestions you have about how our procedures might be improved. Please mention if the timeline for the project seems unreasonable, based on your experiences in the beginning, so that we can re-adjust our expectations and address questions related to budgeting, scheduling, etc. as they arise.

IV. Data Abstraction Overview

- When abstracting and entering data from medical records, it is extremely important to pay careful attention to detail.
  - Be aware of the data abstraction field requirements and complete all fields.
  - Enter data exactly as it is written in the chart, even if you think the chart has an error. If the error in the chart makes it impossible to complete a field (e.g., only 4 digits of a zip code is listed), choose "Not in chart" and then record the data with an explanation in the comments field at the end of the section.
  - Add comments in the final field of each section if you want to communicate something about the form (e.g., lots of missing data, illegible writing, possible error in chart).
  - Take short regular breaks to rest your eyes, stretch and take care of your physical health needs. This helps avoid burnout and data collection errors.
  - If you have any questions, ask!

- For the study, we are recording data in a HIPAA-compliant, web-based database called Qualtrics. You can access the database from any device that is connected to the internet (desktop or laptop computer, iPad). You will be given a study iPad with a keyboard attachment (details in **Section V** below).

- Please give us any feedback about the database early on in the process. UCSF can edit the database during the initial phase of data collection.

- Keep the study iPad safe and secure, as best you can. Do not leave it in plain view when unattended. At the end of each day, **lock the iPad** in a locked office, filing cabinet, or safe. Do not bring the iPad or keyboard attachment off the premises.

V. Data Abstraction Instructions

1. First, affix a green, numbered sticker to the front of every paper chart before entering it into the Qualtrics Database. This 6 digit number will become the patient's Case ID Number. The 1st field in the database requires entering this number. **It is very important that this number is entered accurately**, as it is the link between the physical chart and the information in the database.

2. Then, check to see if the patient had more than one medication abortion during the study period. If they had more than one, only abstract information from the first medication abortion. Make a note in the comments text box of the first section that states: *"Patient had subsequent medication abortion(s) on mm/dd/yyyy".*

3. Begin entering the required data into the Qualtrics database.
   - **Entering data on a laptop or desktop computer:** Make sure your computer is connected to the internet. Open your web browser and enter the link to the Qualtrics database (to be provided at training). It is a good idea to bookmark this link in your web browser since you will be accessing it on a regular basis.

   - **Entering data on the study iPad (with Wi-Fi):** Turn on and unlock the iPad using the passcode (to be provided at training). Make sure the iPad is connected to the clinic Wi-Fi. Find the "**Live Survey – OH MAB"** icon located on the iPad home screen. Click the icon and a blank version of the database will pop up. For each chart, you will click on the icon, so remember to **SUBMIT** the form when you have finished inputting one chart, and close the Safari window, before moving on to the next chart.

4. Enter **all** relevant information from the abortion into the Qualtrics Database using the computer or iPad (**Important: Refer to Appendix A for question-by-question data entry instructions**).

5. The database will automatically skip you to different sets of questions depending on whether the case is from the pre or post-law period.

VI. Quality Assurance

Quality assurance is an extremely important aspect of data entry. We would like each data abstractor to use the ***Data Review Documentation Form*** (**Appendix D**) to compare the data in the database to the data in the actual medical chart on a regular basis. Each sheet of green stickers has one randomly selected sticker with a black dot on it. For each chart with a black-dotted sticker, please do the following:

After data from the black-dotted chart has been entered, but **before submitting the record**, go back to the beginning of the database and compare the data with the original information in the medical chart. Any errors should be corrected in the database and a record should be kept of all errors by question number, using the ***Data Review Documentation Form***. For each error found, repeat the data checking process for the next two consecutive charts and record in the ***Data Review Documentation Form***. This will bring our collective attention to tricky aspects of the data collection process and the database. If there are no or few errors noted after the 200th chart, data checks can occur for every THREE (3) sheets of stickers.

Data collection can be tiring and the process can be imperfect. We expect that there will be some error in the data entry—there always is. Our goal is to minimize the number of errors so that the data analysis is as accurate as possible. Routine data monitoring is a normal aspect of research and is not meant to be punitive. It can be very helpful to refine the data collection tools and processes. In addition, being able to report that we took such measures strengthens the credibility of the study in the eyes of the research audience.

Appendix A: Table of Data Elements with Data Entry Instructions

Data Element	Response Choices	Select only one/Select all that apply	Data Abstraction Instructions
**Abstraction Information**			**Required Section**
0. CASE ID	[Numeric field]		**Must be 6 digits**
1. Site of service	[redacted]	Select only one	If [redacted], skip to 3. If [redacted], continue to 2.
2. Chart Number	[Numeric field]		Positive, whole numbers<br>**Note: This field is for [redacted] only.**
3. Date of abstraction	MM/DD/YYYY		Enter today's date<br>Must be: 8/18/2014 or later
4. Abstractor's name	[Text field]		Enter your name
**Patient Socio-Demographics**			**Required Section**
5. Date of birth	MM/DD/YYYY<br>Not in chart		Format for date<br>Must be: 1/1/1964-12/31/2004<br><br>**Note: All data elements with response choices are required. For missing data, select "Not in chart".**
6. Patient zip code	[Numeric field]<br>Not in chart	Select only one	Limit to 5 digit zip code

Data Element	Response Choices	Select only one/Select all that apply	Data Abstraction Instructions
7. Insurance status	Private insurance<br>Medicaid<br>Medicare<br>Other<br>None<br>Not in chart	Select all that apply	**Note: Record patient insurance status if documented. Do not mark how they paid for their abortion.**
8. Latina/Hispanic	Hispanic<br>Non-Hispanic<br>Not in chart	Select only one	
9. Race	White/Caucasian<br>Black/African American<br>Asian<br>American Indian/Alaskan<br>Pacific Islander<br>Multi/Bi-Racial<br>Other<br>Not in chart	Select all that apply	
10. Highest grade completed	8th grade or less<br>9th-12th grade/No diploma<br>High School Diploma or GED<br>Associates/Some college<br>Bachelor's degree<br>Post Graduate degree<br>Not in chart	Select only one	
11. Height	_______ ft ______inches<br>Not in chart	Select only one	Whole numbers
12. Weight	__________lbs<br>__________kilos<br>Not in chart	Select only one	Whole numbers<br>**Note: Record patient weight as documented. If both pounds and kilos are in the chart, record both.**
13. Body Mass Index (BMI)	__________<br>Not in chart	Select only one	Whole numbers

Data Element	Response Choices	Select only one/Select all that apply	Data Abstraction Instructions
14. Number of previous pregnancies	________<br>Not in chart	Select only one	Whole numbers<br><br>[if "0" skip to 18]<br><br>**Note: Record patient pregnancy history as documented in the chart even if the birth outcomes do not add up to total pregnancies.**
15. Number of previous miscarriages	________<br>Not in chart	Select only one	Whole numbers
16. Number of previous induced abortions	________<br>Not in chart	Select only one	Whole numbers
17. Number of previous births	________<br>Not in chart	Select only one	Whole numbers
a. Number of C-sections	________<br>Not in chart	Select only one	Whole numbers
b. Number of vaginal births	________<br>Not in chart	Select only one	Whole numbers
18. General notes about socio-demographics	[Text field]		

Data Element	Response Choices	Select only one/Select all that apply	Data Abstraction Instructions
**First Consent visit**			**Required Section**
19. Date of first visit	MM/DD/YYYY<br>Not in chart		Format for date
20. Gestational age			
a. Clinical estimate of gestation at consent visit (weeks) – LMP	(_ _ weeks and _ days)<br>(_ days)<br>Not in chart	Select only one	Positive, whole numbers<br><br>If both 20a and 20b are greater than 7 weeks, 0 days <u>or</u> 49 days <u>or</u> not in chart at consent visit, you can stop entering data after Question 20. Close the browser window and start abstracting the next chart.<br><br>**Note: Some sites calculate gestational age by Last Menstrual Period (LMP), other sites calculate gestational age by ultrasound, and some use both and calculate a Final gestational age. Complete 20a through 20c based on standard practice at your site. Complete as many fields as there are data available.**
b. Clinical estimate of gestation at consent visit (weeks) – ULTRASOUND	(_ _ weeks and _ days)<br>(_ days)<br>Not in chart	Select only one	Positive, whole numbers
c. Clinical estimate of gestation at consent visit (weeks) – FINAL	(_ _ weeks and _ days)<br>(_ days)<br>Not in chart	Select only one	Positive, whole numbers<br><br>**Note: "FINAL" is a field mainly used at** [redacted]

Data Element	Response Choices	Select only one/Select all that apply	Data Abstraction Instructions
d. Notes about gestational age	[Text field]		
21. Serum hCG level	________ hCG level<br>Test done but level not available in chart<br>Not in chart	Select only one	Positive, whole numbers
22. a. Urine pregnancy test result	Positive<br>Negative<br>Test done but results not in chart<br>Not in chart	Select only one	
b. Sensitivity of test	High<br>Low<br>Not in chart	Select only one	
23. General notes about consent visit	[Text field]		
**Mifepristone Visit**			
24. Gestational age<br>a. Clinical estimate of gestation at mifepristone visit (weeks) – LMP	(__ __ weeks and __ days)<br>Not in chart	Select only one	Positive, whole numbers<br><br>If both 24a and 24b are greater than 7 weeks, 0 days <u>or</u> 49 days <u>or</u> not in chart at consent visit, you can stop entering data after Question 24. Close the browser window and start abstracting the next chart.<br><br>**Note: Some sites calculate gestational age by Last Menstrual Period (LMP), other sites calculate gestational age by ultrasound, and some use both and calculate a Final**

Data Element	Response Choices	Select only one/Select all that apply	Data Abstraction Instructions
			**gestational age. Complete 20a through 20c based on standard practice at your site. Complete as many fields as there are data available.**
b. Clinical estimate of gestation at mifepristone visit (weeks) – ULTRASOUND	(__ __ weeks and __ days)<br>Not in chart	Select only one	Positive, whole numbers
c. Clinical estimate of gestation at mifepristone visit (weeks) – FINAL	(__ __ weeks and __ days)<br>Not in chart	Select only one	Positive, whole numbers<br><br>**Note: "FINAL" is a field mainly used at [redacted]**
d. Notes about gestational age	[Text field]		
25. Serum hCG level	________ hCG level<br>Test done but level not available in chart<br>Not in chart	Select only one	Positive, whole numbers
26. Urine pregnancy test result	Positive<br>Negative<br>Test done but results Not in chart<br>Not in chart	Select only one	
a. Sensitivity of test	High<br>Low<br>Not in chart	Select only one	

Data Element	Response Choices	Select only one/Select all that apply	Data Abstraction Instructions
27. Date of mifepristone administration	MM/DD/YYYY<br>Not in chart	Select only one	Format for date
a. Time of mifepristone administration	____:____(am/pm)<br>Time not indicated in chart	Select only one	Format for time
28. Mifepristone dose	200 mg<br>600 mg (FDA Protocol)<br>Other (specify) [Text field]<br>Not in chart	Select only one	Limit to positive, whole numbers
29. Other medications ordered, dispensed or administered (Include pain meds, antibiotics and other meds. Do not include contraception. Select all that apply.)	A. Acetaminophen<br>B. Advil<br>C. Amoxicillin<br>D. Azithromycin<br>E. Diazepam<br>F. Doxycycline<br>G. Hydrocodone & Acetaminophen<br>H. Ibuprofen<br>I. Methergine<br>J. Methylergometrine<br>K. MICRhoGam<br>L. Motrin<br>M. Ondansetron<br>N. Phenergan<br>O. Promethazine<br>P. Tylenol<br>Q. Valium<br>R. Vicodin<br>S. Zithromax<br>T. Zofran	Select all that apply	**Note: Include pain meds, antibiotics and other medications. Do not include contraception.**

Data Element	Response Choices	Select only one/Select all that apply	Data Abstraction Instructions
	U. Other (specify) [Text field]<br>V. Not in chart		
30. General notes about Mifepristone visit	[Text field]		**Note here if additional dose of mifepristone was required at this visit and explain.**
**Misoprostol Administration (Pre law change)**			
Did abortion occur in Pre or Post law period?	Pre-law<br>Post-law	Select only one	**Note: One option must be selected. If abortion was started 1/1/2010 – 1/31/2011, select Pre-law. If abortion was started 2/1/2011 or later, select Post-law.**<br><br>If "Post-law", skip to 36.
31. Did expulsion occur after mifepristone alone?	Yes (explain how expulsion was confirmed) [Text field]<br>No<br>Not in chart	Select only one	**Note: Information for this section will usually appear on the form for the first follow up visit.**
32. Date of misoprostol administration	MM/DD/YYYY<br>Not in chart	Select only one	Format for date
33. Time of misoprostol administration	____:____(am/pm)<br>Time not indicated	Select only one	Format for time
34. Method of administration	Oral<br>buccal<br>vaginal<br>other (explain) [Text field]<br>Not in chart	Select only one	**Note: Enter method of administration reported by patient if available.**

Data Element	Response Choices	Select only one/Select all that apply	Data Abstraction Instructions
35. General notes about Misoprostol administration	[Text field]		
**Misoprostol Visit (Post law change)**			
36. Did expulsion occur after mifepristone alone?	Yes (explain how expulsion was confirmed) [Text field]<br>No<br>Not in chart	Select only one	
37. Date of misoprostol administration	MM/DD/YYYY<br>Not in chart	Select only one	Format for date
38. Time of misoprostol administration	____:____(am/pm)<br>Time not indicated	Select only one	Format for time
39. Bleeding since last visit:			[redacted]: **complete 39a and, if available, 39b.**<br><br>[redacted]: **complete 38b only.**
a. Amount of bleeding?	None<br>Minimal<br>Moderate<br>Heavy/Excessive<br>Not in chart	Select only one	**Important:** **Where there is no check box for "None", mark "Not in chart".**<br><br>**Mark "Heavy" or "Excessive" depending on terminology used at your site.**
b. Notes	[Text field]		
40. Cramping since last visit:			[redacted]: **complete 40a and, if appropriate, 40b.**<br><br>[redacted]: **complete 40b only.**

Data Element	Response Choices	Select only one/Select all that apply	Data Abstraction Instructions
a. Amount of cramping?	None<br>Minimal<br>Tolerable<br>Unbearable<br>Not in chart	Select only one	**Important:** **Where there is no check box for "None", mark "Not in chart".**
b. Notes	[Text field]		
41. Other side effects or symptoms experienced since last visit	A. Nausea<br>B. Vomiting<br>C. Diarrhea<br>D. Vasovagal response / Fainting<br>E. Fever<br>F. Chills<br>G. Other (specify) [Text field]<br>H. Not in chart	Select all that apply	**Note: Document any side effects that the patient has experienced at any time since the mifepristone visit. Side effects/ symptoms may be documented in provider notes in chart. If no other side effects/symptoms recorded, select "Not in chart".**
42. Serum hCG level	________ hCG level<br>Test done but level not available in chart<br>Not in chart	Select only one	Positive, whole numbers
43. a. Urine pregnancy test result	Positive<br>Negative<br>Test done but results Not in chart<br>Not in chart	Select only one	
b. Sensitivity of test	High<br>Low<br>Not in chart	Select only one	

Data Element	Response Choices	Select only one/Select all that apply	Data Abstraction Instructions
44. Other medications ordered, dispensed or administered	A. Acetaminophen<br>B. Advil<br>C. Amoxicillin<br>D. Azithromycin<br>E. Diazepam<br>F. Doxycycline<br>G. Hydrocodone & Acetaminophen<br>H. Ibuprofen<br>I. Methergine<br>J. Methylergometrine<br>K. MICRhoGam<br>L. Motrin<br>M. Ondansetron<br>N. Phenergan<br>O. Promethazine<br>P. Tylenol<br>Q. Valium<br>R. Vicodin<br>S. Zithromax<br>T. Zofran<br>U. Other (specify) [Text field]<br>V. Not in chart	Select all that apply	**Note: Include pain meds, antibiotics and other meds. Do not include contraception.**
45. General notes about Misoprostol Visit	[Text field]		

Data Element	Response Choices	Select only one/Select all that apply	Data Abstraction Instructions
**Follow Up Visit**			
46. Total number of follow up visits to complete abortion care	Numeric field		Positive, whole numbers 0-10.<br><br>Important: **Please document the number of follow up visits needed to complete abortion care. Do not include follow up visits for other reasons (i.e. treatment for positive STI test, contraceptive visit, etc.)**
47. Date of follow up visit	MM/DD/YYYY<br>Not in chart	Select only one	Format for date
48. Ultrasound performed at this visit?	Yes<br>No<br>Not in chart	Select only one	If "No" or "Not in chart", skip to 49.
a. Pregnancy sac seen?	No sac seen<br>Sac seen – unknown viability<br>Sac seen – viable pregnancy<br>Sac seen – not viable<br>Possible sac seen<br>Not in chart	Select only one	If "No sac seen" or "Not in chart", skip to 48e.
b. If sac seen: Any notes about a change in the sac over time? Is it persistent or has it grown over time? (Please be as detailed as possible)	[Text field]<br>Not in chart	Select only one	**Note: Please transcribe word-for-word any notes made about changes in how the pregnancy sac looks. Please be as detailed as possible.**

Data Element	Response Choices	Select only one/Select all that apply	Data Abstraction Instructions
c. Embryo seen?	Yes<br>No<br>Not in chart	Select only one	
d. Heartbeat seen?	Yes<br>No<br>Not in chart	Select only one	
e. Notes about amount of material in endometrial cavity with clots or tissue	[Text field]<br>Not in chart	Select only one	
f. Notes on ultrasound findings	[Text field]<br>Not in chart	Select only one	
49. Serum hCG level	________ hCG level<br>Test done but level not available in chart<br>Not in chart	Select only one	Positive, whole numbers
50. a. Urine pregnancy test result	Positive<br>Negative<br>Test done but results not in chart<br>Not in chart	Select only one	If "Not in chart", skip to 51.
b. Sensitivity of test	High<br>Low<br>Not in chart	Select only one	

Data Element	Response Choices	Select only one/Select all that apply	Data Abstraction Instructions
51. Current bleeding:			**[redacted]: complete 51a and, if appropriate, 51b.**<br><br>**[redacted]: complete 51b only.**
a. Amount of bleeding?	None<br>Minimal/Light<br>Moderate<br>Heavy<br>Not in chart	Select only one	**Important: Where there is no check box for "None", mark "Not in chart".**<br><br>**Mark "Minimal" or "Light" depending on terminology used at your site.**
b. Notes on bleeding	[Text field]		
52. Notes on cramping	[Text field]		
53. Other side effects or symptoms experienced since last visit (mark all that apply)	A. Nausea<br>B. Vomiting<br>C. Diarrhea<br>D. Vasovagal response / Fainting<br>E. Fever<br>F. Chills<br>G. Other (specify)<br>H. Not in chart	Select all that apply	If G=yes, continue to 53a.<br><br>For all others, skip to 54.<br><br>**Note: Document any side effects that the patient has experienced at any time since the mifepristone visit. Other side effects/symptoms may be documented in provider notes in chart. If no other side effects/symptoms recorded, select "Not in chart".**
a. Specify other symptoms	[Text field]<br>Not in chart		

Data Element	Response Choices	Select only one/Select all that apply	Data Abstraction Instructions
54. Any notes recorded about how and when the pregnancy was passed? (i.e. In the car on the way home, how long after the miso it was passed)	[Text field]<br>Not in chart	Select only one	**Note: Please transcribe word-for-word**
55. Diagnosis at follow up visit	Mark all that apply:<br>A. Complete abortion<br>B. Possible incomplete Abortion<br>C. Incomplete Abortion<br>D. Continuing Pregnancy<br>E. Acute Hemorrhage<br>F. Infection<br>G. Other or Undetermined (please explain) [Text field]<br>H. No Diagnosis Given (please explain) [Text field]<br>I. Not in chart	Select all that apply<br><br>[cannot mark I if A-H are marked]	If G and/or H = yes, continue to 55a.<br><br>If D=yes skip to 56.<br><br>For all others, skip to 57.
If continuing pregnancy detected:			Complete only if 55D = yes
56. Continuing pregnancy detected by ultrasound?	Yes<br>No<br>Not in chart	Select only one	Complete only if 55D = yes
a. Continuing	Yes	Select only one	Complete only if 55D = yes

Data Element	Response Choices	Select only one/Select all that apply	Data Abstraction Instructions
pregnancy confirmed by serum hCG level?	No<br>Not in chart		
57. Is there any indication that the patient requested intervention?	Yes<br>No<br>Not in chart	Select only one	
58. Intervention provided at this visit?	Yes<br>No<br>Not in chart	Select only one	If "No" or "Not in chart" skip to 59.
a. Please explain what intervention was provided	[Text field]		**Note: Provide a summary of all intervention provided at this visit, including medications dispensed, ordered, or administered. Please be as detailed as possible.**
59.<br>a. Treatment at this visit at the abortion facility	A. Repeat misoprostol<br>B. Aspiration<br>C. Repeat mife+miso<br>D. Blood transfusion<br>E. Hospital transfer<br>F. Medication<br>G. Other treatment (Specify) [Text field]<br>H. Not in chart	Select all that apply	If F=yes, continue to 59b<br><br>For all others, skip to 60
b. Specify medication	A. Acetaminophen<br>B. Advil<br>C. Amoxicillin<br>D. Azithromycin<br>E. Diazepam	Select all that apply	**Note: Include pain meds, antibiotics and other meds. Do not include contraception. Doses not needed.**

Data Element	Response Choices	Select only one/Select all that apply	Data Abstraction Instructions
	F. Doxycycline<br>G. Hydrocodone & Acetaminophen<br>H. Ibuprofen<br>I. Methergine<br>J. Methylergometrine<br>K. MICRhoGam<br>L. Motrin<br>M. Ondansetron<br>N. Phenergan<br>O. Promethazine<br>P. Tylenol<br>Q. Valium<br>R. Vicodin<br>S. Zithromax<br>T. Zofran<br>U. Other [Text field]<br>V. Not in chart		
60. a. Patient treated at another facility?	A. Yes, Urgent Care<br>B. Yes, Emergency Department<br>C. Yes, Hospital<br>D. No<br>E. Not in Chart	Check all that apply	If "No" or "Not in Chart" skip to 61<br><br>Note: If A, B, or C=yes, then cannot mark D or E and vice versa.
b. Treatment at urgent care/ED/hospital	A. Repeat misoprostol<br>B. Aspiration<br>C. Repeat mife+miso<br>D. Blood transfusion<br>E. Hospitalization (admission)<br>F. Medication (Specify)<br>G. IV fluids	Select all that apply	If F = yes, skip to 60c.<br><br>For all others, skip to 61d

Data Element	Response Choices	Select only one/Select all that apply	Data Abstraction Instructions
	H. No treatment, observation only Other treatment (Specify) [Text field]<br>I. Not in chart		
c. Specify medication	A. Acetaminophen<br>B. Advil<br>C. Amoxicillin<br>D. Azithromycin<br>E. Diazepam<br>F. Doxycycline<br>G. Hydrocodone & Acetaminophen<br>H. Ibuprofen<br>I. Methergine<br>J. Methylergometrine<br>K. MICRhoGam<br>L. Motrin<br>M. Ondansetron<br>N. Phenergan<br>O. Promethazine<br>P. Tylenol<br>Q. Valium<br>R. Vicodin<br>S. Zithromax<br>T. Zofran<br>U. Other [Text field]<br>V. Not in chart		**Note: Include pain meds, antibiotics and other meds. Do not include contraception.**
d. Notes treatment at other facility	[Text field]		

Data Element	Response Choices	Select only one/Select all that apply	Data Abstraction Instructions
61. General notes about this follow up visit	[Text field]		
**Additional Information / Care**			
62. Additional ultrasounds	Yes<br>No<br>Not in chart	Select only one	If "No" or "Not in chart", skip to 63<br><br>**Additional ultrasound visits may take place that are unrelated to the medication abortion (i.e. ruling out ectopic pregnancy, ultrasound related to fetal heartbeat law). Note all additional ultrasounds and associated dates.**
a. Date of additional ultrasound(s)	MM/DD/YYYY<br>Not in chart	Select only one	Format for date<br><br>Allow for entering of up to 10 dates
b. Notes about additional ultrasound	[Text field]<br>Not in chart		Repeat for each additional date
63. Medical Board RU-486 Report, "Remarks" Section	[Text field]		**Note: A copy of the Medical Board RU-486 Report should be in every chart in which additional intervention or complication occurred. Please transcribe "Remarks" word-for-word.**
64. Care sought at outside provider?	Yes<br>No<br>Not in chart	Select only one	If "No" or "Not in chart", skip to 65<br><br>**If care was sought/received at any outside provider(s) during the course of the abortion (emergency room, urgent care, family doctor, etc.), please record associated date(s) and notes about where care what sought and any treatment provided.**

Data Element	Response Choices	Select only one/Select all that apply	Data Abstraction Instructions
a. Date(s)	MM/DD/YYYY<br>Not in chart	Select only one	Format for date<br><br>Allow for entering of up to 10 dates
b. Notes about care sought at outside provider	[Text field]<br>Not in chart		Repeat for each additional date<br><br>**Document where the patient went and how she was treated.**
65. Calls to after-hours line?	Yes<br>No<br>Not in chart	Select only one	If "No" or "Not in chart", skip to 66<br><br>**Document all calls to after-hours line and associated date(s).**
a. Dates	MM/DD/YYYY<br>Not in chart	Select only one	Format for date<br><br>Allow for entering of up to 10 dates
b. Notes about afterhours calls	[Text field]<br>Not in chart	Select only one	Repeat for each additional date<br><br>**Provide summary or transcribe call notes word-for word.**
66. Relevant test results (blood)	[Text field]		**Document the results of any additional, relevant blood tests.**
67. General Notes	[Text field]		**Record any additional notes about the abortion and/or chart that do not fit anywhere else in this form.**

Appendix B: FDA vs. Evidence-Based Protocol for Medication Abortion

	FDA regimen	Updated regimen: Vaginal Misoprostol	Updated regimen: Buccal Misoprostol
**Maximum gestational age**	49 days from LMP	63 days from LMP	63 days from LMP
**Mifepristone dose**	600 mg. orally in office	200 mg. orally in office	200 mg. orally in office
**Misoprostol dose**	400 mcg orally (2 tablets)	800mcg. vaginally (4 tablets)	800 mcg. buccally (4 tablets)
**Misoprostol timing**	48 hours after mifepristone	6-72 hours after mifepristone	24-36 hours after mifepristone
**Misoprostol location**	Clinician's office	Home	Home
**Follow-up visit**	14 days after mifepristone	7-14 days after mifepristone	7-14 days after mifepristone
**Minimum number of office visits in Ohio**	4	3	3

Source: National Abortion Federation

Appendix C: Visit Flowcharts

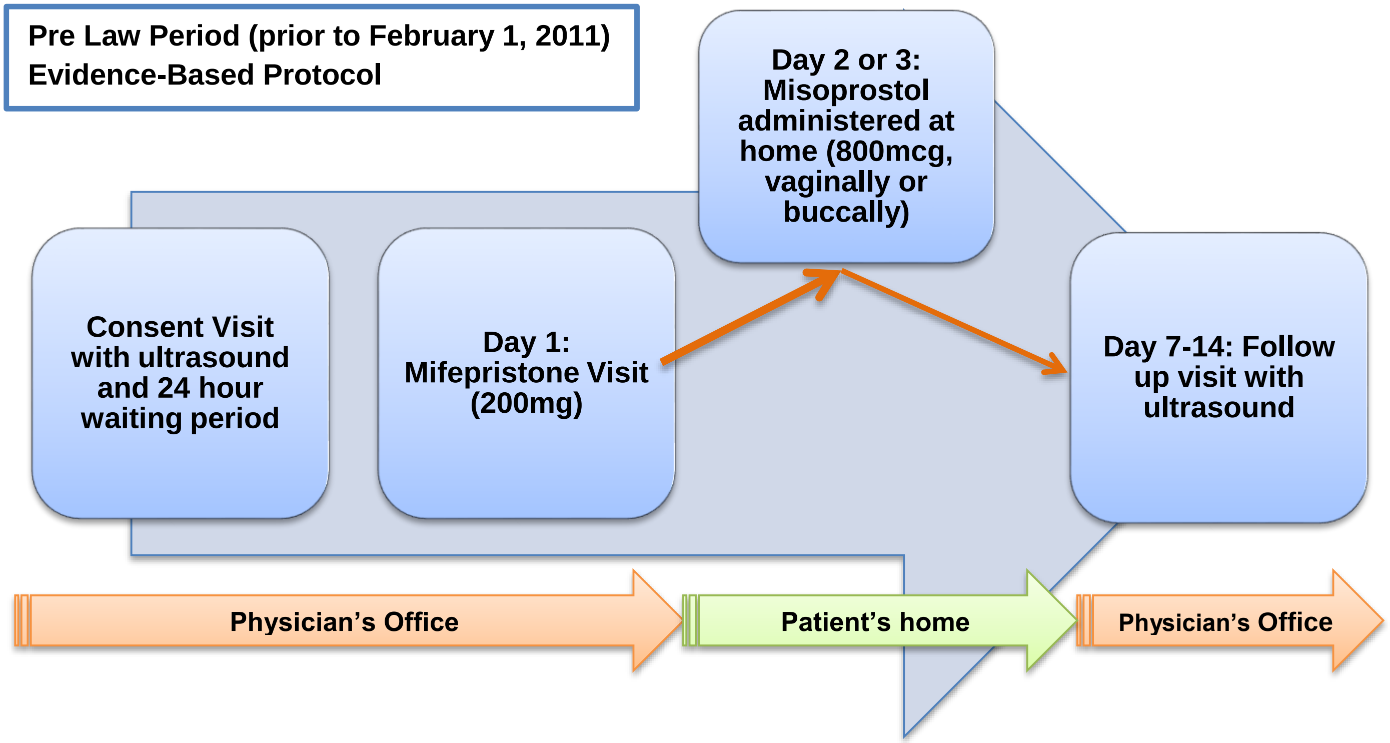

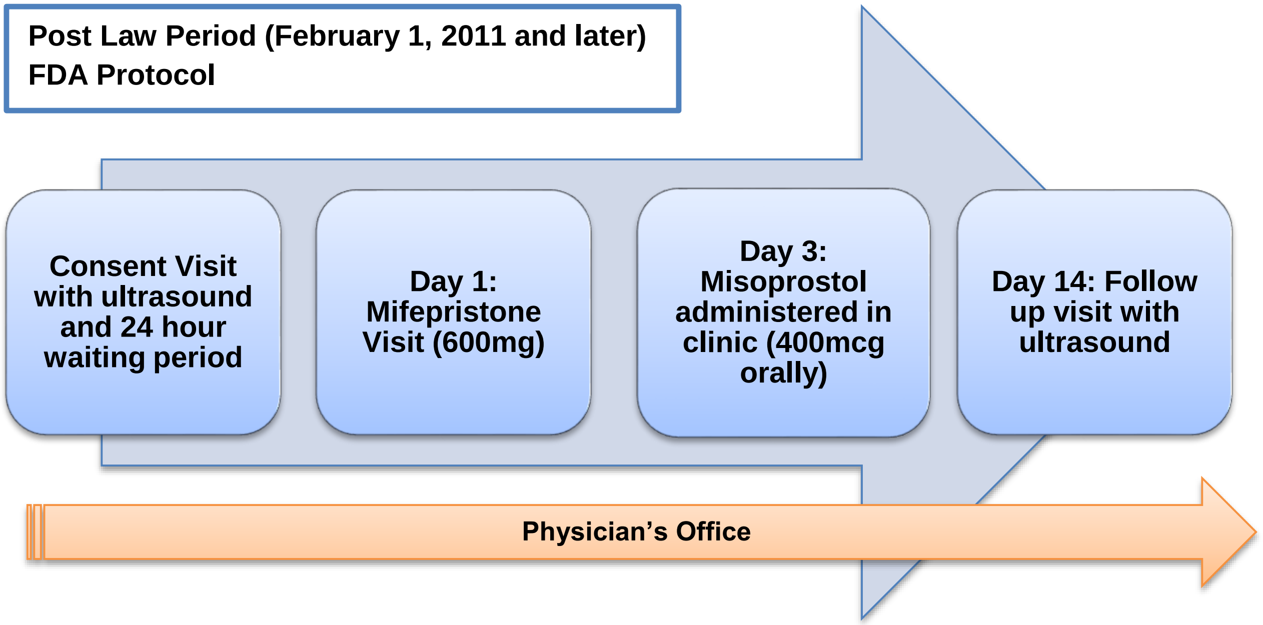

Appendix D: Data Review Documentation Form

Please use this form to document data review of charts marked with a black dot.

Record ID	Error(s) NO	Error(s) YES	Data Field	Incorrect Entry(ies)	Corrections Made	Initials
05-0001	*X*					*SC*
05-0030		*X*	*Date of birth*	*5/20/1980*	*5/20/1989*	*SC*
05-0031	*X*					*SC*
05-0032	*X*					*SC*

Record ID	Error(s) NO	Error(s) YES	Data Field	Incorrect Entry(ies)	Corrections Made	Initials

Record ID	Error(s) NO	Error(s) YES	Data Field	Incorrect Entry(ies)	Corrections Made	Initials

Record ID	Error(s) NO	Error(s) YES	Data Field	Incorrect Entry(ies)	Corrections Made	Initials

Record ID	Error(s) NO	Error(s) YES	Data Field	Incorrect Entry(ies)	Corrections Made	Initials

Record ID	Error(s) NO	Error(s) YES	Data Field	Incorrect Entry(ies)	Corrections Made	Initials

Record ID	Error(s) NO	Error(s) YES	Data Field	Incorrect Entry(ies)	Corrections Made	Initials

Record ID	Error(s) NO	Error(s) YES	Data Field	Incorrect Entry(ies)	Corrections Made	Initials

Record ID	Error(s) NO	Error(s) YES	Data Field	Incorrect Entry(ies)	Corrections Made	Initials

Record ID	Error(s) NO	Error(s) YES	Data Field	Incorrect Entry(ies)	Corrections Made	Initials

Record ID	Error(s) NO	Error(s) YES	Data Field	Incorrect Entry(ies)	Corrections Made	Initials

Record ID	Error(s) NO	Error(s) YES	Data Field	Incorrect Entry(ies)	Corrections Made	Initials

Record ID	Error(s) NO	Error(s) YES	Data Field	Incorrect Entry(ies)	Corrections Made	Initials

Record ID	Error(s) NO	Error(s) YES	Data Field	Incorrect Entry(ies)	Corrections Made	Initials

Record ID	Error(s) NO	Error(s) YES	Data Field	Incorrect Entry(ies)	Corrections Made	Initials

Record ID	Error(s) NO	Error(s) YES	Data Field	Incorrect Entry(ies)	Corrections Made	Initials
